# Bivariate Causal Discovery and Its Applications to Gene Expression and Imaging Data Analysis

**DOI:** 10.3389/fgene.2018.00347

**Published:** 2018-08-31

**Authors:** Rong Jiao, Nan Lin, Zixin Hu, David A. Bennett, Li Jin, Momiao Xiong

**Affiliations:** ^1^Department of Biostatistics and Data Science, The University of Texas School of Public Health, Houston, TX, United States; ^2^Ministry of Education Key Laboratory of Contemporary Anthropology, School of Life Sciences, Fudan University, Shanghai, China; ^3^Rush Alzheimer's Disease Center, Rush University Medical Center, Chicago, IL, United States; ^4^Human Phenome Institute, Fudan University, Shanghai, China

**Keywords:** independence of cause and mechanism (ICM), algorithmic information theory, additive noise models, genome-wide causal studies, gene expression, imaging data analysis

## Abstract

The mainstream of research in genetics, epigenetics, and imaging data analysis focuses on statistical association or exploring statistical dependence between variables. Despite their significant progresses in genetic research, understanding the etiology and mechanism of complex phenotypes remains elusive. Using association analysis as a major analytical platform for the complex data analysis is a key issue that hampers the theoretic development of genomic science and its application in practice. Causal inference is an essential component for the discovery of mechanical relationships among complex phenotypes. Many researchers suggest making the transition from association to causation. Despite its fundamental role in science, engineering, and biomedicine, the traditional methods for causal inference require at least three variables. However, quantitative genetic analysis such as QTL, eQTL, mQTL, and genomic-imaging data analysis requires exploring the causal relationships between two variables. This paper will focus on bivariate causal discovery with continuous variables. We will introduce independence of cause and mechanism (ICM) as a basic principle for causal inference, algorithmic information theory and additive noise model (ANM) as major tools for bivariate causal discovery. Large-scale simulations will be performed to evaluate the feasibility of the ANM for bivariate causal discovery. To further evaluate their performance for causal inference, the ANM will be applied to the construction of gene regulatory networks. Also, the ANM will be applied to trait-imaging data analysis to illustrate three scenarios: presence of both causation and association, presence of association while absence of causation, and presence of causation, while lack of association between two variables. Telling cause from effect between two continuous variables from observational data is one of the fundamental and challenging problems in omics and imaging data analysis. Our preliminary simulations and real data analysis will show that the ANMs will be one of choice for bivariate causal discovery in genomic and imaging data analysis.

## Introduction

Despite significant progress in dissecting the genetic architecture of complex diseases by association analysis, understanding the etiology, and mechanism of complex diseases remains elusive. Using association analysis and machine learning systems that operate, almost exclusively, in a statistical, or model-free modes as a major analytic platform for genetic studies of complex diseases is a key issue that hampers the discovery of mechanisms underlying complex traits (Pearl, 2018).

As an alternative to association analysis, causal inference may provide tools for unraveling principles underlying complex traits. Causation is defined as the act that generates an effect in Merriam-Webster dictionary. In terms of daily life language, causation is the effects of actions or interventions that perturb the system or indicates that one event is the result of the occurrence of the other event. In statistics, the causal effect can be defined using intervention calculus (Mooij et al., 2016). Suppose that *X, Y* are two random variables with joint distribution *P*(*X, Y*). If an external intervention that is from outside the system under consideration forces the variable *X* to have the value *x* and keeps the rest of the system unchanged, after *Y* is measured, the resulting distribution of *Y*, *P*(*Y*|*do* (*x*)) is defined as the causal effect of *X* on *Y*. Power of causal inference is its ability to predict effects of actions on the system (Mooij et al., 2016).

Typical methods for unraveling cause-and-effect relationships are interventions and controlled experiments. Unfortunately, the experiments in human genetics are unethical and technically impossible. Next generation genomic, epigenomic, sensing, and image technologies produce ever deeper multiple omic, physiological, imaging, environmental, and phenotypic data with millions of features. These data are almost all “observational,” which have not been randomized or otherwise experimentally controlled (Glymour, 2015). In the past decades, a variety of statistical methods and computational algorithms for causal inference which attempt to abstract causal knowledge from purely observational data, referred to as causal discovery, have been developed (Zhang et al., 2018). Causal inference is one of the most useful tools developed in the past century. The classical causal inference theory explores conditional independence relationships in the data to discover causal structures. The PC algorithms and the fast causal inference (FCI) algorithms developed at Carnegie Mellon University by Peter Spirtes and Clark Glymour are often used for cause discovery (Le et al., 2016). Despite its fundamental role in science, engineering and biomedicine, the conditional independence-based classical causal inference methods can only identify the graph up to its Markov equivalence class, which consists of all directed acyclic graphs (DAGs) satisfying the same conditional independence distributions via the causal Markov conditions (Nowzohour and Bühlmann, 2016). DAGs are defined as directed graphics with no cycles. In other words, we can never start at a node, travel edges in the directions of the arrows and get back to the node (Figure S1). For example, consider three simple DAGs: *x* → *y* → *z*, *x* ← *y* ← *z*, and *x* ← *y* → *z*. Three variables *x, y*, and *z* in all three DAGs satisfy the same causal Markov condition: *x* and *z* are independent, given *y*. This indicates that these three DAGs form a Markov equivalence class. However, these three DAGs represent three different causal relationships among variables *x, y*, and *z*, which prohibits unique causal identification. These non-unique causal solutions seriously limit their translational application.

In the past decade, causal inference theory is undergoing exciting and profound changes from discovering only up to the Markov equivalent class to identify unique causal structure (Peters et al., 2011; Peters and Bühlman, 2014). A class of powerful algorithms for finding a unique causal solution is based on properly defined functional causal models (FCMs). They include the linear, non-Gaussian, acyclic model (LiNGAM) (Shimizu et al., 2006; Zhang et al., 2018), the additive noise model (ANM) (Hoyer et al., 2009; Peters et al., 2014), and the post-nonlinear (PNL) causal model (Zhang and Hyvärinen, 2009).

In genomic and epigenomic data analysis, we usually consider four types of associations: association of discrete variables (DNA variation) with continuous variables (quantitative trait, gene expressions, methylations, imaging signals and physiological traits), association of continuous variables (expressions, methylations and imaging signals) with continuous variables (gene expressions, imaging signals, phenotypes and physiological traits), association of discrete variables (DNA variation) with binary trait (disease status), and association of continuous variables (gene expressions, methylations, phenotypes and imaging signals) with binary trait (disease status). All these four types of associations can be extended to four types of causations. This paper focuses on studying causal relationships between two continuous variables.

Many causal inference algorithms using observational data require that two variables being considered as cause-effect relationships are part of a larger set of observational variables (Mooij et al., 2016). Similar to genome-wide association studies where only two variables are considered, we mainly investigate bivariate causal discovery to infer cause-effect relationships between two observed variables. To simplify the causal discovery studies, we assume no selection bias, no feedback and no confounding. We first introduce the basic principle underlying the modern causal theory. It assumes that nature consists of autonomous and independent causal generating process modules and attempts to replace causal faithfulness (If every conditional independence in the distribution is implied by the Markov condition in the DAG, it requires that every variables is independent of its non-descendants in the DAG) by the assumption of Independence of Cause and Mechanism (ICM) (Janzing and Schölkopf, 2010; Schölkopf et al., 2012; Lemeire and Janzing, 2013; Besserve et al., 2017; Peters et al., 2017). Then, we will present ANM as a major tool for causal discovery between two continuous variables. We will investigate properties of ANM for causal discovery. Finally, the ANM will be applied to gene expression data to infer gene regulatory networks and longitudinal phenotype-imaging data to identify brain regions affected by intermediate phenotypes. A program for implementing the algorithm for bivariate causal discovery with two continuous variables can be downloaded from our website https://sph.uth.edu/research/centers/hgc/xiong/software.htm.

## The independence principle of cause and mechanism for causal inference

This section will introduce the independence of cause and mechanism as a basic principle for causal inference and Kolmogorov complexity as a theoretic tool for causal analysis. The philosophical causal principle assumes that nature consists of independent, autonomous causal generating process modules (Shajarisales et al., 2015; Peters et al., 2017). In other words, causal generating processes of a system's variables are independent. If we consider two variables: cause *X* and effect *Y*, then the mechanism that generates cause *X* and the mechanism that generates effect *Y* from the cause *X* are independent. Or, the process that generates the effect *Y* from the cause *X* contains no information about the process that generates the cause *X*. In the probability setting, this indicates that the cause distribution *P*(*X*) and the conditional distribution *P*(*Y*|*X*) of *Y* given *X* are independent. Statistics provides definition of independence between two random variables, but provides no tools for defining independence between two distributions (Peters et al., 2017). Algorithmic information theory can offer notion and mathematical formulation of independence between two distributions or independence of mechanisms (Janzing and Schölkopf, 2010; Parascandolo et al., 2017).

Cause and effect cannot be identified from their joint distribution. Cause and effect are asymmetric. The joint distribution is symmetric. It can be factorized to *P*_*X, Y*_ = *P*_*X*_*P*_*Y*|*X*_ = *P*_*Y*_*P*_*X*|*Y*_. (For details, please see Supplementary Note A). This implies that the joint distribution *P*_*X, Y*_ of two variables *X, Y* is unable to infer whether *X* → *Y* or *Y* → *X*. Peters et al. (2014) showed in Proposition 4.1 of their book that for every joint distribution *P*_*X, Y*_ or *P*_*Y, X*_ of real-valued variables *X* and *Y*, there are non-linear models:

$$Y=f_{Y}\left(X,N_{Y}\right),X_{⫫}N_{Y}$$

and

$$X=g_{X}\left(Y,N_{X}\right),Y_{⫫}N_{X},$$

where *f*_*Y*_ and *g*_*X*_ are functions and *N*_*Y*_ and *N*_*X*_ are real-valued noise variables. In Supplementary Note B we provide the details that were omitted in the proof of Proposition 4.1 (Peters et al., 2014). This shows that to make a bivariate causal model identifiable, we must restrict the function class.

## Non-linear additive noise models for bivariate causal discovery

In this section, we will introduce popular non-linear additive noise models (ANMs) as a major tool for bivariate causal discovery. Assume no confounding, no selection bias and no feedback. Consider a bivariate additive noise model *X* → *Y* where *Y* is a non-linear function of *X* and independent additive noise *E*_*Y*_:

(1) $$\begin{array}{lll} Y & = & f_{Y}\left(X\right)+E_{Y}\\ X & \sim & P_{X},E_{Y}\sim P_{E_{Y}}, \end{array}$$

where *X* and *E*_*Y*_ are independent. Then, the density *P*_*X, Y*_ is said to be induced by the additive noise model (ANM) from *X* to *Y* (Mooij et al., 2016). The alternative additive noise model between *X* and *Y* is the additive noise model *Y* → *X*:

(2) $$\begin{array}{lll} X & = & f_{X}\left(Y\right)+E_{X}\\ Y & \sim & P_{Y},E_{X}\sim P_{E_{X}}, \end{array}$$

where *Y* and *E*_*X*_ are independent.

If the density *P*_*X, Y*_ is induced by the ANM *X* → *Y*, but not by the ANM *Y* → *X*, then the ANM *X* → *Y* is identifiable. To illustrate application of the algorithmic mutual information, we show that independence of cause and mechanism will imply that the cause *X* and error *E*_*Y*_ in the non-linear function model (1) are independent (For details, please see Supplementary Note C).

**Figure 1:**
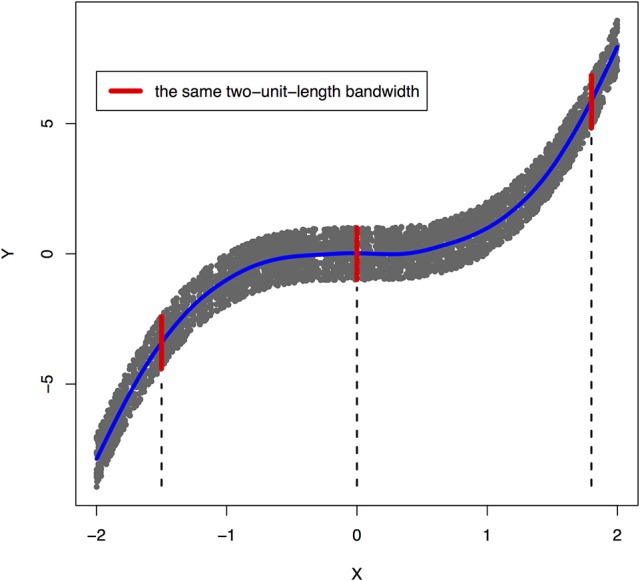
An example of joint distribution *p*(*x, y*) generated by *Y*: = *f*(*X*) + *E*_*Y*_, where *f*(*X*) = *X*^3^ and *E*_*Y*_ is uniformly distributed in [−1, 1]. The interval of the red line represents the bandwidth of the conditional distribution *p*_*Y*|*X*_. We perform a nonlinear regression in the directions *X* → *Y*.

**Figure 2:**
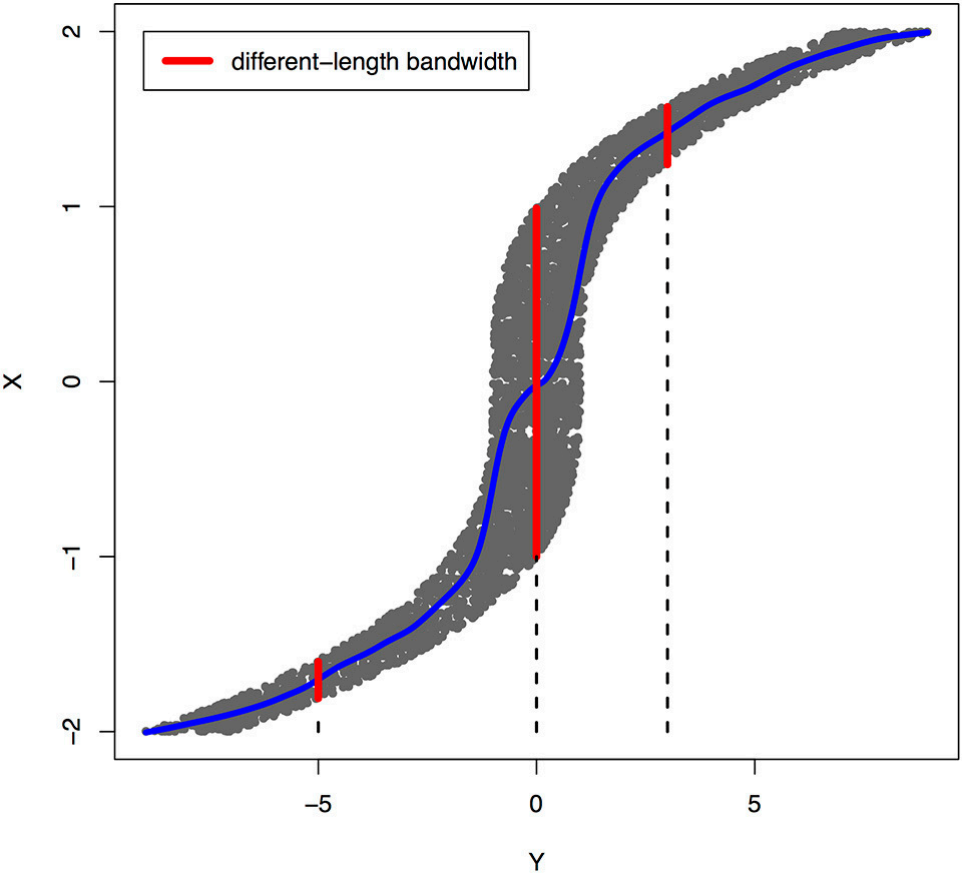
An example of joint distribution *p*(*x, y*) generated by *Y*: = *f*(*X*) + *E*_*Y*_, where *f*(*X*) = *X*^3^ and *E*_*Y*_ is uniformly distributed in [−1, 1]. The interval of the red line represents the bandwidth of the conditional distribution *p*_*X*|*Y*_. We perform a nonlinear regression in the directions *Y* → *X*.

Peters et al. (2017) showed that a joint distribution *P*_*X, Y*_ does not admit an ANM in both directions at the same time under some quite generic conditions. To illustrate that ANMs are generally identifiable, i.e., a joint distribution only admits an ANM in one direction, we plotted Figures 1, 2. The data in Figures 1, 2 were generated by $Y=X^{3}+E_{Y}$, where *E*_*Y*_ is uniformly distributed in [−1, 1].

The joint distribution satisfied an ANM *X* → *Y*, but did not admit an ANM *Y* → *X*. We plotted Figures 1, 2 in which red lines indicated the bandwidth of the conditional distribution. Figure 1 showed that all bandwidth of the conditional distribution *P*_*Y*|*X*_ represented by the red line was two units. This clearly demonstrated that conditional distribution *P*_*Y*|*X*_ did not depend on the cause *X*. However, Figure 2 showed that the bandwidth of the conditional distribution *P*_*X*|*Y*_, represented by the red line varied as *Y* changed. This demonstrated that the conditional distribution *P*_*X*|*Y*_, indeed, depended on *Y*. In other words, it violated the principal of independence of cause and mechanism. The joint distribution in this example only admitted an ANM in only one direction *X* → *Y*.

The ANMs should assume that the functions *f*_*X*_ and *g*_*Y*_ are non-linear. If the functions are linear, then additional assumptions for identifiability should be made. In other words, for the linear functions, if at least one of the distributions of the cause and noise is non-Gaussian [e.g., linear non-Gaussian acyclic model (LiNGAM)], then the linear model is identifiable. Otherwise, the linear model is not identifiable (Shimizu et al., 2011; Moneta et al., 2013). In this scenario, we cannot get different bandwidths. The limitation of the ANMs is that it cannot be applied to linear case if both distributions of cause and noise are Gaussian.

Empirically, if the ANM *X* → *Y* fits the data, then we infer that *X* causes *Y*, or if the ANM *Y* → *X* fits the data, then *Y* causes *X* will be concluded. Although this statement cannot be rigorously proved, in practice, this principle will provide the basis for bivariate cause discovery (Mooij et al., 2016). To implement this principal, we need to develop statistical methods for assessing whether the additive noise model fits the data or not.

Now we summarize procedures for using ANM to assess causal relationships between two variables. Two variables can be two gene expressions, or one gene expression and one methylation level of CpG site, or an imaging signal of one brain region and a functional principal score of gene. Divide the dataset into a training data set by specifying $D_{train}=\left\{Y_{n},X_{n}\right\},Y_{n}={\left[y_{1},\ldots ,y_{n}\right]}^{T},X_{n}={\left[x_{1},\ldots ,x_{n}\right]}^{T}$ for fitting the model and a test data set $D_{test}=\left\{{\tilde{Y}}_{m},{\tilde{X}}_{m}\right\},{\tilde{Y}}_{m}={\left[{\tilde{y}}_{1},\ldots ,{\tilde{y}}_{m}\right]}^{T},{\tilde{X}}_{m}={\left[{\tilde{x}}_{1},\ldots ,{\tilde{x}}_{m}\right]}^{T}$ for testing the independence, where *n* is not necessarily equal to *m*.

*Algorithm for causal discovery with two continuous variables is given below*.

Step 1. Regress *Y* on *X* using the training dataset *D*_*train*_ and non-parametric regression methods:
  (3) $$\begin{array}{l} Y=\hat{f}\left(X\right)+E_{Y}. \end{array}$$
Step 2. Calculate residual ${\tilde{E}}_{Y}=Y-\hat{f}\left(X\right)$ using the test dataset *D*_*test*_ and test whether the residual Ê_*Y*_ is independent of causal *X* to assess the ANM *X* → *Y*.
Step 3. Repeat the procedure to assess the ANM *Y* → *X*.
Step 4. If the ANM in one direction is accepted and the ANM in the other is rejected, then the former is inferred as the causal direction.

There are many non-parametric methods that can be used to regress *Y* on *X* or regress *X* on *Y*. For example, we can use smoothing spline regression methods (Wang, 2011), B-spline (Wang and Yan, 2017) and local polynomial regression (LOESS, see Cleveland, 1979).

Covariance can be used to measure association, but cannot be used to test independence between two variables. A covariance operator can measure the magnitude of dependence, and is a useful tool for assessing dependence between variables. Specifically, we will use the Hilbert-Schmidt norm of the cross-covariance operator or its approximation, the Hilbert-Schmidt independence criterion (HSIC) to measure the degree of dependence between the residuals and potential causal variable (Gretton et al., 2005; Mooij et al., 2016).

*Calculation of the HSIC consists of the following steps*.

Step 1: Use test data set to compute
  $$\begin{array}{l} y_{i}=\hat{f}\left(x_{i}\right)+E_{Y}\left(i\right),i=1,\ldots ,m. \end{array}$$
Step 2: Compute the residuals:
  $$\begin{array}{l} \varepsilon_{i}=E_{Y}\left(i\right)=y_{i}-\hat{f}\left(x_{i}\right),=1,\ldots ,m. \end{array}$$
Step 3: Select two kernel functions *k*_*E*_(ε_*i*_, ε_*j*_) and *k*_*x*_(*x*_1_, *x*_2_). In practice, we often use the Gaussian kernel function. Compute the Kernel matrices:
  $$\begin{array}{l} K_{E_{Y}}=\left[\begin{array}{ccc} k_{E}\left(\varepsilon_{1},\varepsilon_{1}\right) & \ldots & k_{E}\left(\varepsilon_{1},\varepsilon_{m}\right)\\ \vdots & \vdots & \vdots \\ k_{E}\left(\varepsilon_{m},\varepsilon_{1}\right) & \ldots & k_{E}\left(\varepsilon_{m},\varepsilon_{m}\right) \end{array}\right],K_{x}=\left[\begin{array}{ccc} k_{x}\left(x_{1},x_{1}\right) & \ldots & k_{x}\left(x_{1},x_{m}\right)\\ \vdots & \vdots & \vdots \\ k_{x}\left(x_{m},x_{1}\right) & \ldots & k_{x}\left(x_{m},x_{m}\right) \end{array}\right]. \end{array}$$
Step 4: Compute the HSCI for measuring dependence between the residuals and potential causal variable.
  $$\begin{array}{l} HSIC^{2}\left(E_{Y},X\right)=\frac{1}{m^{2}}Tr\;\left(K_{E_{Y}}HK_{X}H\right), \end{array}$$
  where $H=I-\frac{1}{m}1_{m}1_{m}^{T},1_{m}={\left[1,1,\ldots ,1\right]}^{T}$ and Tr denotes the trace of the matrix.

*In summary, the general procedure for bivariate causal discovery is given as follows* (Mooij et al., 2016):

Step 1: Divide a data set into a training data set *D*_*train*_ = {*Y*_*n*_, *X*_*n*_} for fitting the model and a test data set $D_{test}=\left\{{\tilde{Y}}_{m},{\tilde{X}}_{m}\right\}$ for testing the independence.
Step 2: Use the training data set and non-parametric regression methods
  - Regress *Y* on *X*: *Y* = *f*_*Y*_(*X*) + *E*_*Y*_ and
  - Regress *X* on *Y*: *X* = *f*_*X*_(*X*) + *E*_*X*_.
Step 3: Use the test data set and estimated non-parametric regression model that fits the training data set *D*_*train*_ = {*Y*_*n*_, *X*_*n*_} to predict residuals:
  - ${\tilde{E}}_{Y_{X}}=\tilde{Y}-{\hat{f}}_{Y}\left(\tilde{X}\right)$
  - ${\tilde{E}}_{X_{Y}}=\tilde{X}-{\hat{f}}_{X}\left(\tilde{Y}\right)$.
Step 4: Calculate the dependence measures $HSIC^{2}\left(E_{Y},X\right)$ and $HSIC^{2}\left(E_{X},Y\right)$.
Step 5: Infer causal direction:
  (4) $$\begin{array}{l} X\to Y\;if\;HSIC^{2}\left(E_{Y},X\right)<HSIC^{2}\left(E_{X},Y\right); \end{array}$$
  (5) $$\begin{array}{l} Y\to X\;if\;HSIC^{2}\left(E_{Y},X\right)>HSIC^{2}\left(E_{X},Y\right). \end{array}$$
  If $HSIC^{2}\left(E_{Y},X\right)=HSIC^{2}\left(E_{X},Y\right)$, then causal direction is undecided.

We do not have closed analytical forms for the asymptotic null distribution of the HSIC and hence it is difficult to calculate the *P*-values of the independence tests. To overcome these limitations, the permutation/bootstrap approach can be used to calculate the *P*-values of the causal test statistics. The null hypothesis is

*H*_0_: no causations *X* → *Y* and *Y* → *X* (Both *X* and *E*_*Y*_ are dependent, and *Y* and *E*_*X*_ are dependent).

Calculate the test statistic:

(6) $$\begin{array}{l} T_{C}=|HSIC^{2}\left(E_{Y},X\right)-HSIC^{2}\left(E_{X},Y\right)|. \end{array}$$

Assume that the total number of permutations is *n*_*p*_. For each permutation, we fix *x*_*i*_, *i* = 1, …, *m* and randomly permutate *y*_*i*_, *i* = 1, …, *m*. Then, fit the ANMs and calculate the residuals *E*_*X*_(*i*), *E*_*Y*_(*i*), *i* = 1, …, *m* and test statistic *T*_*C*_. Repeat *n*_*p*_ times. The *P*-values are defined as the proportions of the statistic ${\tilde{T}}_{C}$ (computed on the permuted data) greater than or equal to ${\hat{T}}_{C}$ (computed on the original data*D*_*TE*_). After cause is identified, we then use Equations (4) and (5) to infer causal directions *X* → *Y* or *Y* → *X*.

## Linear correlation and causation

In everyday language, correlation and association are used interchangeably. However, correlation and association are different terminologies. Pear correlation coefficient is defined as $\rho =\frac{cov\left(X,Y\right)}{\sigma_{x}\sigma_{y}}$ from covariance, Spearman correlation coefficient is defined as measuring increasing or decreasing trends. Association characterizes dependence between two variables (Altman and Krzywinski, 2015). In this paper, association is equivalent to Pearson linear correlation. We will focus on linear correlation. We investigate the relationships between causation and correlation. The correlation between two continuous variables can be investigated by a linear regression model:

(7) $$\begin{array}{l} Y=\beta X+\varepsilon , \end{array}$$

where β≠0.

The causation *X* → *Y* is identified by the ANM:

(8) Y=f(X)+ε,X⫫ε.

In classical statistics, if we assume that both variables *X* and ε follow a normal distribution, then *cov*(*X*, ε) = 0 if and only if *X* and ε are independent. If *X* and ε are not normal variables, this statement will not hold. For general distribution, we extend the concept of covariance to cross covariance operator ${\tilde{C}}_{X\varepsilon }$ (Zhang et al., 2018). It is shown that for the general distributions of *X* and ε, ${\tilde{C}}_{X\varepsilon }=0$ if and only if *X* and *Y* are independent (Mooij et al., 2016).

Let *h* and *g* be any two non-linear functions. ${\tilde{C}}_{X\varepsilon }=0$ is equivalent to (Gretton et al., 2005)

(9) $$\begin{array}{l} \max cov\left(h\left(X\right),g\left(\varepsilon \right)\right)=\max cov\left(h\left(X\right),g\left(Y-f\left(X\right)\right)\right)=0, \end{array}$$

Subject to ||*h*|| = 1, ||*g*|| = 1.

Now we give examples of a pair of random variables to illustrate existence of three cases: (a) both linear correlation and causation *X* → *Y*, (b) causation *X* → *Y*, but no linear correlation, and (c) linear correlation, but no causation *X* → *Y*.

*a) Both linear correlation and causation X* → *Y*.

We consider a special case: *Y* = *f*(*X*). When *Y* = *f*(*X*), Equation (9) holds, which implies *X* → *Y*. If we assume that *h*(*X*) = *X* and *g*(*Y* − *f*(*X*)) = *Y* − *f*(*X*), then Equation (9) holds and implies that

(10) $$\begin{array}{l} cov\left(X,Y\right)=cov\left(X,f\left(X\right)\right). \end{array}$$

If we further assume *f*(*X*) = β*X*, then Equation (10) implies

(11) $$\begin{array}{l} \beta =\frac{cov\left(X,Y\right)}{Var\left(X\right)}. \end{array}$$

This is estimation of linear regression coefficient.

*b) Causation* *X* → *Y, but no linear correlation*

Consider the model:

$$\begin{array}{l} Y=5X^{2}+\varepsilon , \end{array}$$

where *X* follows a uniform distribution between −2 and 2 and ε follows a uniform distribution between −1 and 1.

**Figure 3:**
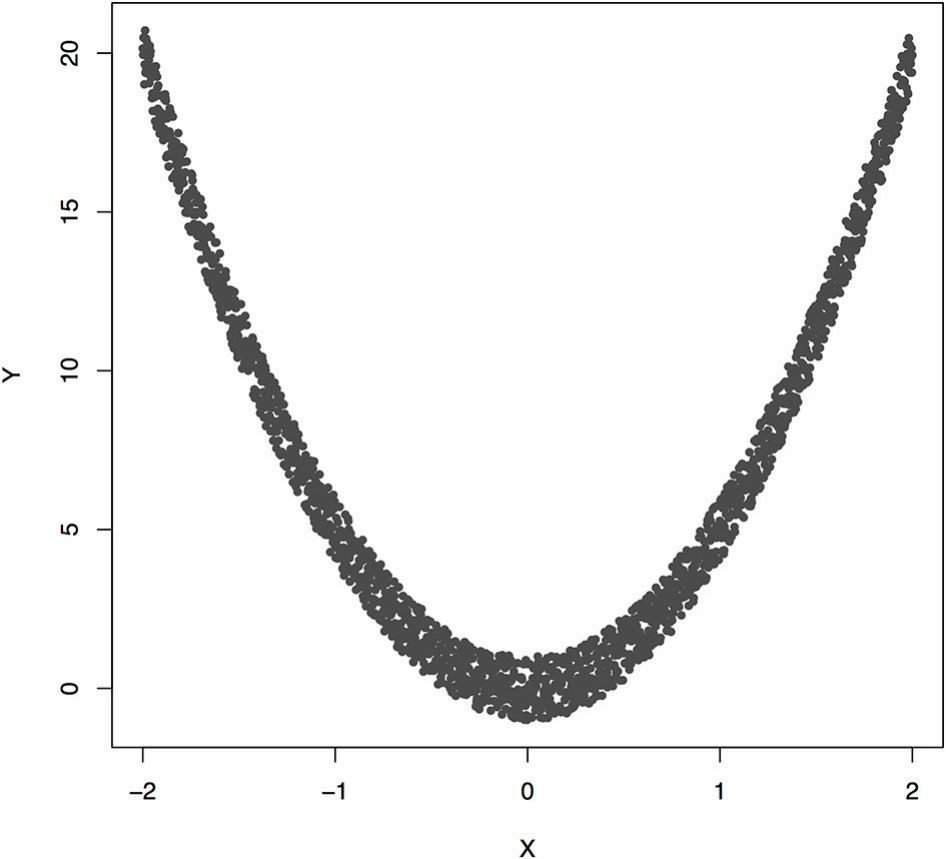
The data generated by *Y* = 5*X*^2^ + ε, where *X* follows a uniform distribution between −2 and 2 and ε follows a uniform distribution between −1 and 1.

Figure 3 plotted functions *Y* = 5*X*^2^ + ε. Assume that 2,000 subjects were sampled. Permutation was used to calculate *P*-value for testing causation. We found that the Pearson correlation was −0.00070 and *P*-value for testing causation *X* → *Y* was 10^−5^. This example showed the presence of causation, but lack of linear correlation (Pearson correlation was near zero).

*c) Linear correlation, but no causation* *X* → *Y*.

Consider the model (Figure S2):

$$\begin{array}{l} X=Z+\varepsilon_{1},\\ Y=Z+\varepsilon_{2}, \end{array}$$

and *Z* ~ *N*(0, 2), ε_1_ ~ *N*(0, 1), ε_2_ ~ *N*(0, 1), *Z*, ϵ_1_, ε_2_ are independent.

The model can be rewritten as

$$\begin{array}{l} Y=X+\varepsilon_{2}-\varepsilon_{1}. \end{array}$$

First we show that linear correlation between *Y* and *X* exists.

In fact,

$$\begin{array}{l} cov\left(Y,X\right)=cov\left(Z+\varepsilon_{2},Z+\varepsilon_{1}\right)=var\left(Z\right)\\ \;=2,Var\left(Y\right)=3,Var\left(X\right)=3. \end{array}$$

Thus, the Pearson linear correlation coefficient is equal to $\rho =\frac{2}{3}$. Thus, linear correlation between *Y* and *X* exists.

Next we show that *X* and ε_2_ − ε_1_ are not independent.

Note that *cov*(*X*, ε_2_ − ε_1_) = −*var*(ε_1_) = −1 and *X*, ε_2_ − ε_1_ follow normal distribution. Since the covariance between *X* and ε_2_ − ε_1_ is not equal to zero, this implies that *X* and ε_2_ − ε_1_ are not independent. The conditional distribution *P*_*Y*|*X*_ is the distribution of ε_2_ − ε_1_. But, we show that the normal variables *X* and ε_2_ − ε_1_ are not independent. This implies that the distribution *P*(*X*) and *P*_*Y*|*X*_ are not independent. Therefore, we finally show that there is no causation *X* → *Y*.

Similar conclusions hold for *Y* → *X*.

## Simulations

### ANMs with different non-linear functions

To investigate their feasibility for causal inference, the ANMs were applied to simulation data. Similar to Nowzohour and Bühlmann (2016), we considered three non-linear functions: quadratic, exponential, and logarithm functions and two random noise variables: normal and *t* distribution. We assumed that the cause *X* follows a normal distribution *N*(0, 1).

First we consider two models with a quadratic function and two types of random noise variables, normal *N*(0, 1) and *t* distribution with 5 degrees of freedom:

Model 1:
  $$\begin{array}{l} Y=X+b\cdot X^{2}+\varepsilon_{1}, \end{array}$$
  where the parameter *b* ranges from −10 to 10 and ε_1_ is distributed as N (0,1).
Model 2:
  $$\begin{array}{l} Y=X+b\cdot X^{2}+\varepsilon_{2} \end{array}$$
  where the parameter *b* is defined as before and ε_2_ is distributed as *t* distribution with 5 degrees of freedom.

**Figure 4:**
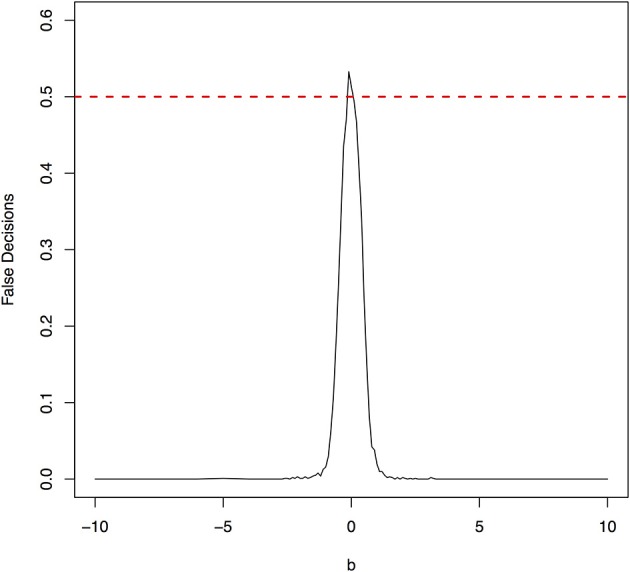
False decision rates as a function of the parameter *b* for the model 1.

**Figure 5:**
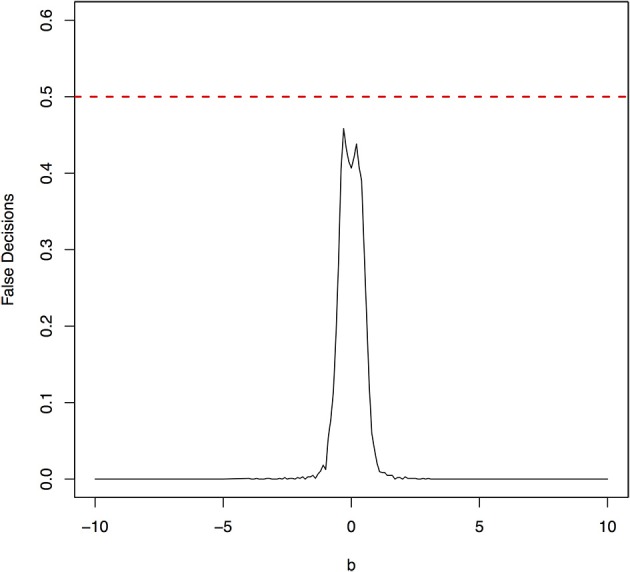
False decision rates as a function of the parameter *b* for the model 2.

The parameter space *b* ∈ [−10, 10] was discretized. For each grid point, 1,000 simulations were repeated. For each simulation, 500 samples were generated. The ANMs were applied to the generated data. Smoothing spline is used to fit the functional model. The true causal direction is the forward model: *X* → *Y*. The false decision rate was defined as the proportion of times when the backward model *Y* → *X* is wrongly chosen by the ANMs. Figures 4, 5 presented false decision rate as a function of the parameter *b* for the models 1 and 2, respectively. We observed from Figures 4, 5 that the false decision rate reached its maximum 0.5 when *b* = 0. This showed that when the model is close to linear, the ANMs could not identify the true causal direction. However, when *b* moved away from 0, the false decision rates approached 0 quickly. This showed that when the data were generated by non-linear models, with high probability, we can accurately identify the true causal directions.

To further confirm these observations, we consider another two non-linear functions.

Model 3:
  $$\begin{array}{l} Y=X+b\log\left(\left|X\right|\right)+\varepsilon_{1}, \end{array}$$
Model 4:
  $$\begin{array}{l} Y=X+b\log\left(\left|X\right|\right)+\varepsilon_{2}, \end{array}$$
Model 5:
  $$\begin{array}{l} Y=X+b\cdot e^{X}+\varepsilon_{1}, \end{array}$$
Model 6:
  $$\begin{array}{l} Y=X+b\cdot e^{X}+\varepsilon_{2}, \end{array}$$
  where the parameter *b* and the noise variables ε_1_ and ε_2_ were defined previously.

**Figure 6:**
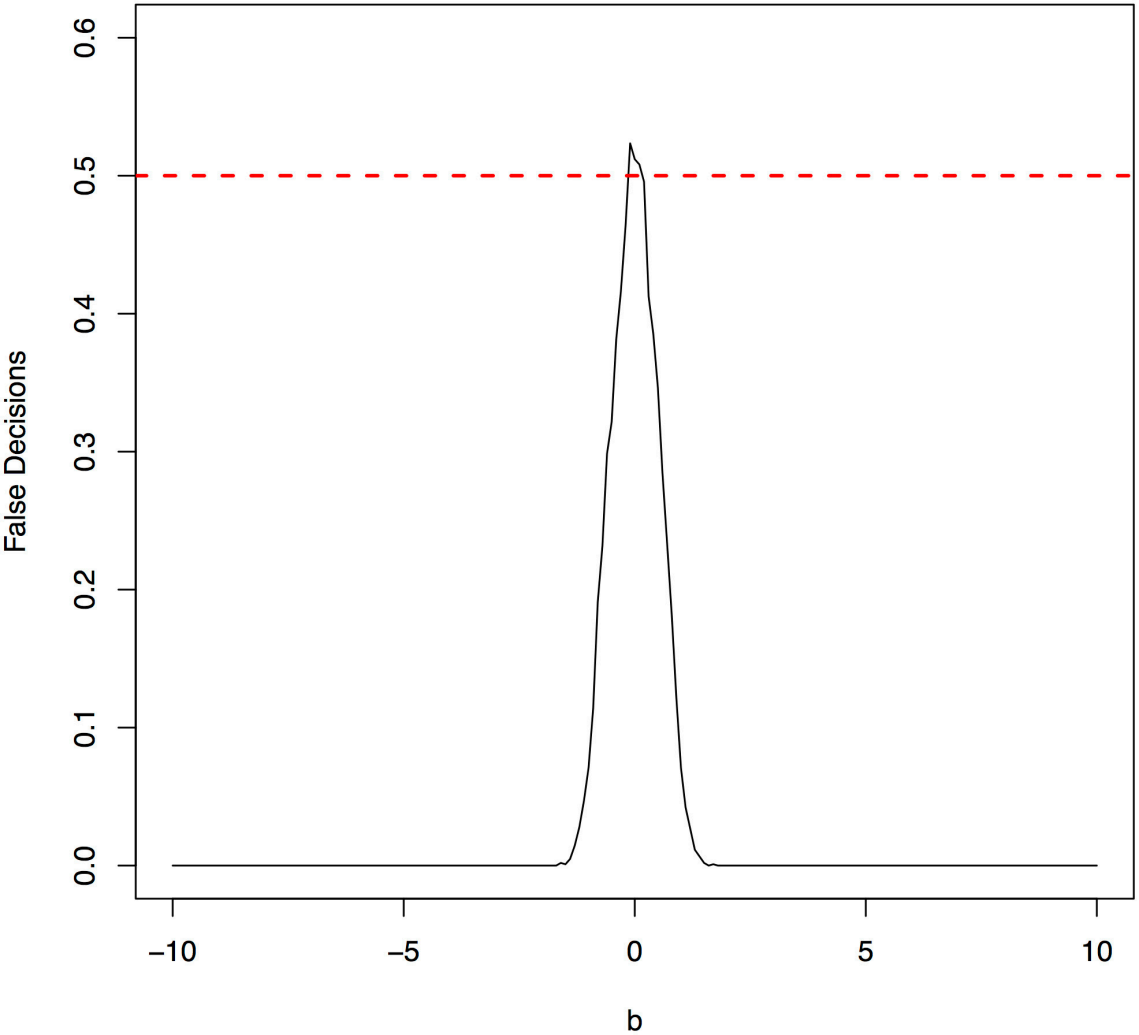
The false decision rates of the ANMs for detecting the true causal direction *X* → *Y* for the model 3.

**Figure 7:**
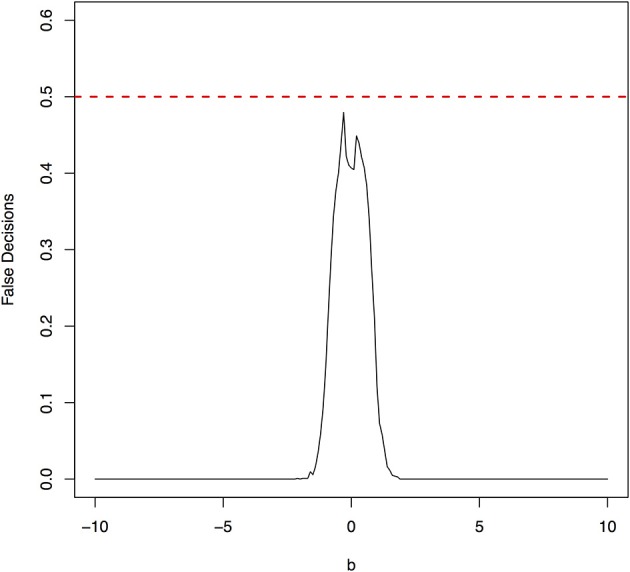
The false decision rates of the ANMs for detecting the true causal direction *X* → *Y* for the model 4.

**Figure 8:**
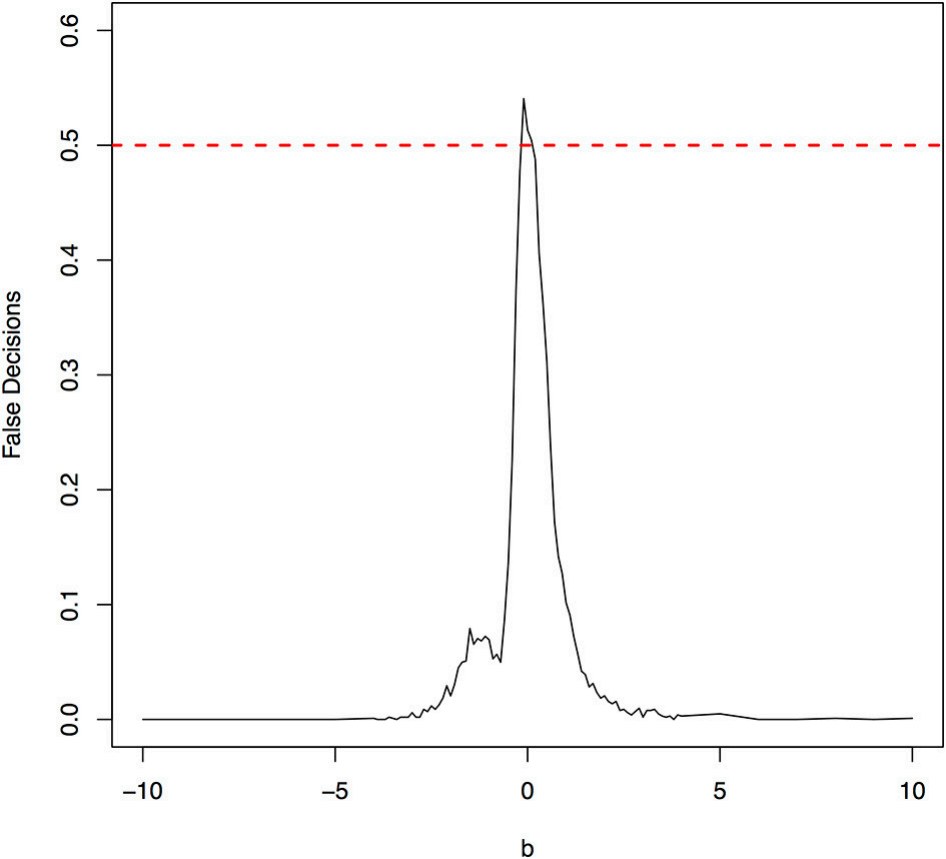
The false decision rates of the ANMs for detecting the true causal direction *X* → *Y* for the model 5.

**Figure 9:**
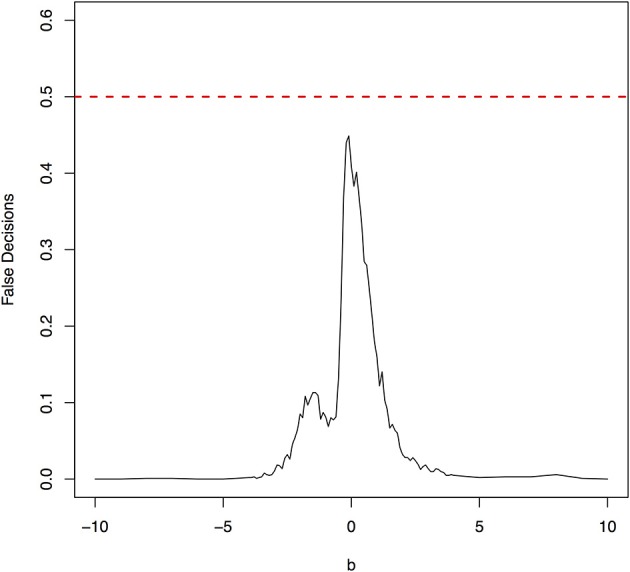
The false decision rates of the ANMs for detecting the true causal direction *X* → *Y* for the model 6.

The false decision rates of the ANMs for detecting the true causal direction *X* → *Y* for the models 3, 4, 5, and 6 were presented in Figures 6–9, respectively. Again, the observations for the models 1 and 2 still held for the models 3, 4, 5, and 6. When the data were generated by non-linear models, we can accurately identify the true causal directions. However, when the data were generated by linear models, the false decision rates reached 0.5, which was equivalent to random guess.

### Type 1 error rates

To evaluate the performance of the ANMs for bivariate cause discovery, we calculate the type 1 error rates. We consider two scenarios: (a) no association, (b) presence of association.

- No association
  We first generated the data with 100,000 subjects from the model: *X* ~ *N*(0, 1), *Y* ~ *N*(0, 1) and *X, Y* are independent. Number of permutations was 500. Number of replication of tests was 1,000. The sampled subjects from the generated population for type 1 error rate calculations were 500, 1,000 and 2,000 respectively. The test statistic *T*_*c*_ and permutations were used to test for causation between two variables *X* and *Y*. Table S1 summarized type 1 error rates of the ANMs for testing causation, assuming no association.
- Presence of association
  Then, we generated the data with 100,000 subjects from the model: *X* ~ *N*(0, 1), *Y* ~ *N*(0, 1), *X* and *Y* were associated, but without causation. Number of permutations was 500. Number of replication of tests was 1,000. The sampled subjects from the generated population for type 1 error rate calculations were 500, 1,000 and 2,000 respectively. The test statistic *T*_*c*_ and permutations were used to test for causation between two variables *X* and *Y*. Table S2 summarized type 1 error rates of the ANMs for testing causation in the presence of association.

In summary, Tables S1, S2 showed that type 1 error rates of the ANM based on permutation even in the presence of association were not significantly deviated from nominal levels.

### Power simulations

To further evaluate the performance of the ANMs for bivariate cause discovery, we used simulated data to estimate their power to detect causation. We generated data with 100,000 subjects from the causal model:

$$\begin{array}{l} Y=f\left(X\right)+\text{N}, \end{array}$$

where $f\left(x\right)=\sum_{j=1}^{3}w_{j}\times exp\left(-\gamma {\left(x-x_{j}\right)}^{2}\right),\gamma \sim N\left(0,1\right)$, *x*_*j*_ ~ *N*(0, 1), *X* ~ *N*(0, 1)and *N* ~ *N*(0, σ^2^ = 0.01). *X* and *N* are independent, and $w_{j}^{\prime }s$ are randomly generated weights from the uniform distribution. Number of permutations was 500. Number of replication of tests was 1,000. The sampled subjects from the population were 200, 500, 1,000, 2,000 and 5,000 respectively. The test statistic *T*_*c*_ and permutations were used to test for causation between two variables *X* and *Y*. Table S3 summarized the power of the ANMs for detecting causation between two variables.

## Real data analysis

Regulation of gene expression is a complex biological process. Large-scale regulatory network inference provides a general framework for comprehensively learning regulatory interactions, understanding the biological activity, devising effective therapeutics, identifying drug targets of complex diseases and discovering the novel pathways. Uncovering and modeling gene regulatory networks are one of the long-standing challenges in genomics and computational biology. Various statistical methods and computational algorithms for network inference have been developed. The ANMs can also be applied to inferring gene regulatory networks using gene expression data. Similar to co-gene expression networks where correlations are often used to measure dependence between two gene expressions, the ANMs can be used to infer regulation direction, i.e., whether changes in expression of gene *X* causes changes in expression of gene *Y* or vise verse changes in expression of gene *Y* causes changes in expression of gene *X*.

The ANMs were applied to Wnt signaling pathway with RNA-Seq of 79 genes measured in 447 tissue samples in the ROSMAP dataset (White et al., 2017). For comparisons, the structural equation models (SEMs) integrating with integer programming (Xiong, 2018), causal additive model (CAM) (Bühlmann et al., 2014), PC algorithm (Tan et al., 2011), random network, glasso (Friedman et al., 2008), and Weighted Correlation Network Analysis (WGCNA) (Langfelder and Horvath, 2008) were also included in the analysis. We ranked directed edges according to the values of the test statistics for the ANMs. The results for top 40, 50, and 60 edges were included in comparison. The results were summarized in Table 1. True directed path was defined as the paths that matched KEGG paths with directions. True undirected path was defined as the paths that matched KEGG paths with or without directions. Detection accuracy was defined as the proportion of the number of true paths detected over the number of all paths detected.

**Table 1:** Accuracy of the ANMs and other six methods for inferring Wnt pathway.

**Wnt pathway**	**Directed paths**			**Undirected paths included**		
**Top selected edge number**	**40**	**50**	**60**	**40**	**50**	**60**
Pairwise ANM	37.50%	38%	35%	47.50%	46%	41.70%
CAM	17.50%	16%	13.30%	25%	24%	25%
SEM	22.50%	20%	15%	32.50%	26%	25%
Random network	25.80%	25.40%	25.40%	31%	30.60%	30.50%
PC algorithm	19.50%	21.60%	16.40%	36.60%	39.20%	27.90%
WGCNA association	X	X	X	25%	22%	23.30%
Glasso	X	X	X	25%	28%	26.70%

**Figure 10:**
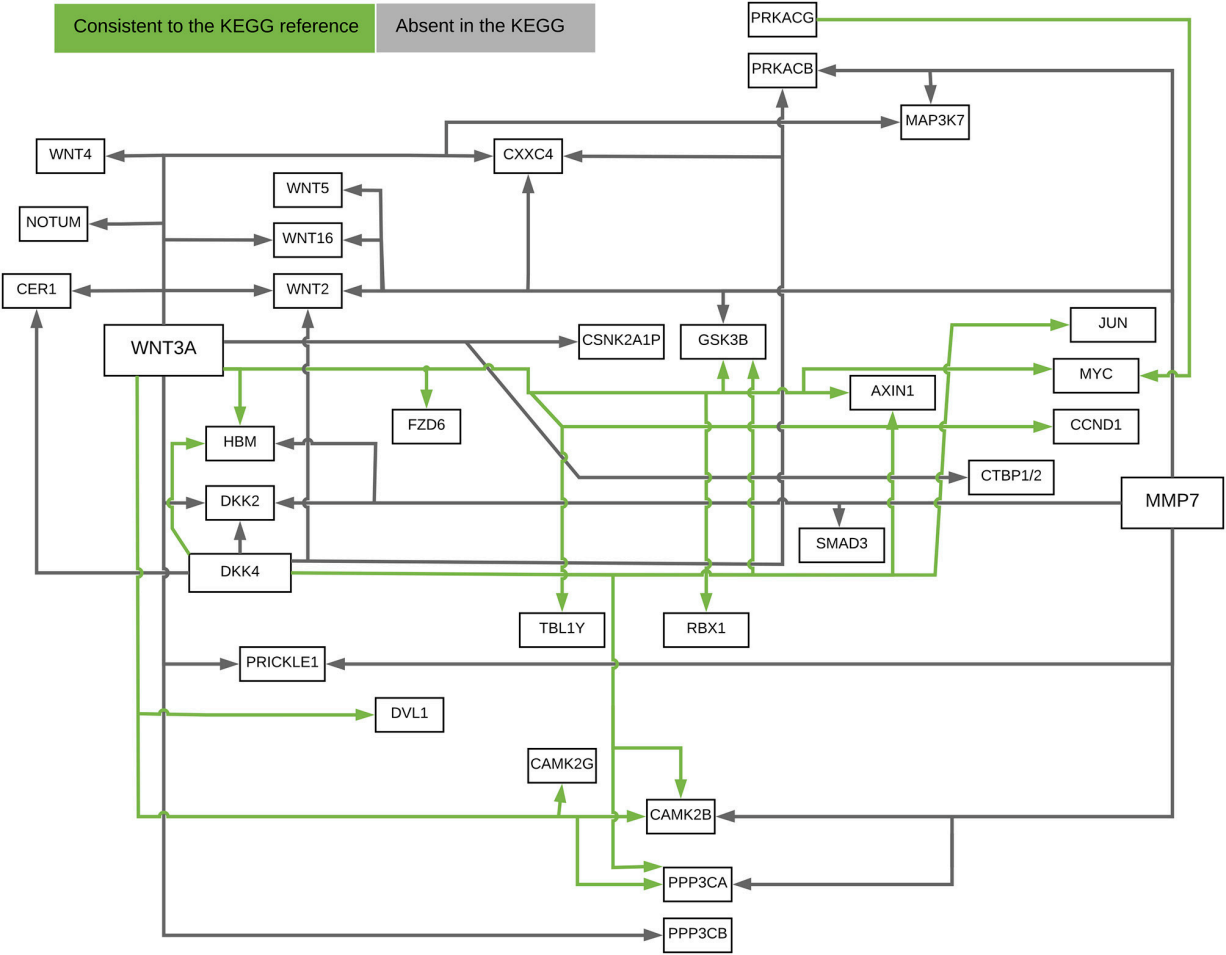
The ANM-inferred network structure of the Wnt pathway. The green lines represented the inferred paths consistent to the KEGG while the gray ones represented the inferred edges absent in the KEGG.

Figure 10 presented the ANM-inferred network structure of the Wnt pathway. The green lines represented the inferred paths consistent to the KEGG while the gray ones represented the inferred edges absent in the KEGG. The ANM, CAM, SEM, PC, and random network methods inferred directed networks, and Glasso and WGCNA association methods inferred undirected networks. We took the structure of Wnt in the KEGG as the true structure of the Wnt in nature. We observed from Table 1 that the ANM more accurately inferred the network structure of the Wnt than the other six statistical and computational methods for identifying directed or undirected networks. Table 1 also showed that the accuracy of widely used Glasso and WGCNA algorithms for identifying the structure of Wnt was even lower than that of random networks, however, the accuracy of the ANM was much higher than that of random networks. The causal network with 50 selected top edges identified by the ANMs reached the highest accuracy. Varying the number of selected edges in the network will affect accuracy, but their accuracies were not largely different for the ANMs. This observation may not be true for other methods.

**Figure 11:**
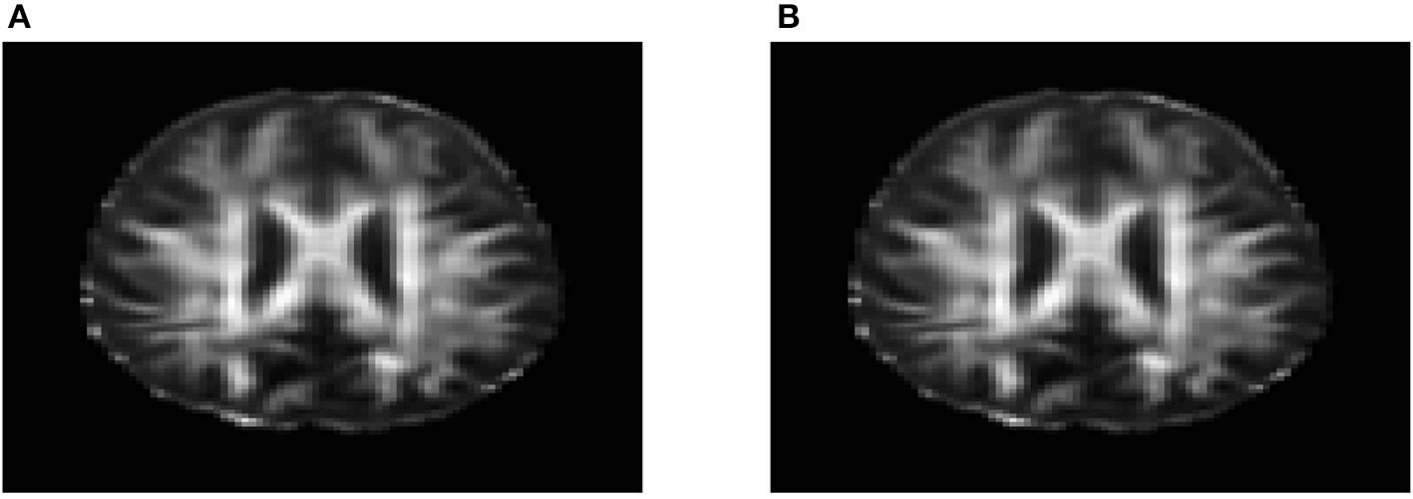
**(A)** A slice of the FA map from a single individual's DTI data. **(B)** FA map reconstruction with the first two 3D-FPC scores.

To evaluate their performance for causal inference, the ANMs were applied to the Alzheimer's Disease Neuroimaging Initiative (ADNI) data with 91 individuals with Diffusion Tensor Imaging (DTI) and cholesterol phenotypes measured at four time points: baseline, 6, 12, and 24 months. After normalization and image registration, the dimension of a single DTI image is 91 × 109 × 91. Three dimensional functional principal component analysis (3D-FPC) was used to summarize imaging signals in the brain region (Lin et al., 2015), because of the technical difficulty and operational cost, only 44 of the 91 individuals have all the DTI imaging data at all the four data points. Based on our own analysis experience, usually the first one or two 3D-FPC scores can explain more that 95% of the variation of the imaging signals in the region. To evaluate the performance of 3D-FPC for imaging signal feature extraction, we present Figures 11A,B. Figure 11A is a layer of the FA map of the DTI image from a single individual and the dimension of this image is 91 × 109. A total of 91 images were used to calculate the 3D-FPC scores. Figure 11B was the reconstruction of the same layer of the FA map of the DTI image from the same individual in Figure 11A using 5 FPC scores. Comparing Figure 11A with Figure 11B, we can see that these two images are very similar indicating that the 3D-FPC score is an effective tool to represent the image features.

**Figure 12:**
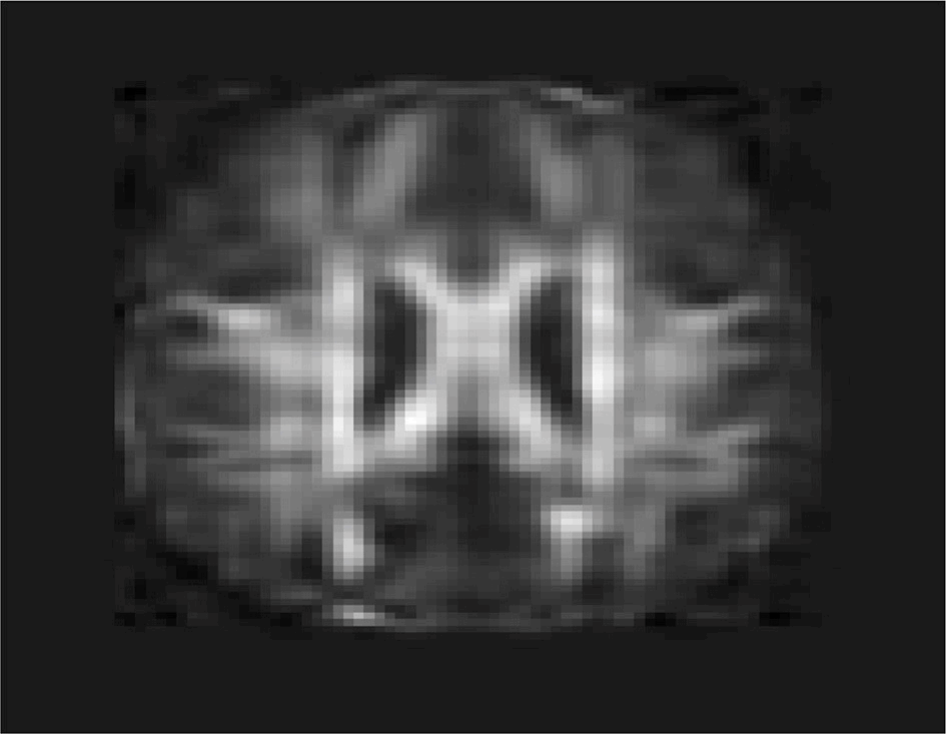
Imputed FA map in Figure 11A using 3D-FPC scores and matrix completion.

To investigate feasibility of image imputation by using a mixed strategy of 3D-FPC scores and matrix completion, we used the DTI image of the 44 individuals who have measurement at all four time points as the investigation dataset. Since at baseline, the DTI image of all individuals was available, we did not have missing value problems. We only need to impute images at 6, 12, and 24 months for some individuals. We randomly sampled 20 individuals assuming that their imaging data were missing. Matrix completion methods were used to impute missing images (Thung et al., 2018). To perform 3D FPCA, all missing imaging signals at 6, 12, and 24 months of the individuals were replaced by their imaging signals at the baseline. Then, 3D FPCA was performed on the original images and replaced images of 44 individuals at all time points (base line, 6, 12, and 24 months). The FPC scores of 22 individuals without missing images were used for matrix completion. The imputed FPC score were then used to form reconstruction of the DTI images. To evaluate performance of the above image imputation, we presented Figure 12 that was the reconstruction of the DTI image in Figure 11A. We observed from these figures that the imputed image captured the majority of the information in the original DTI image data.

**Figure 13:**
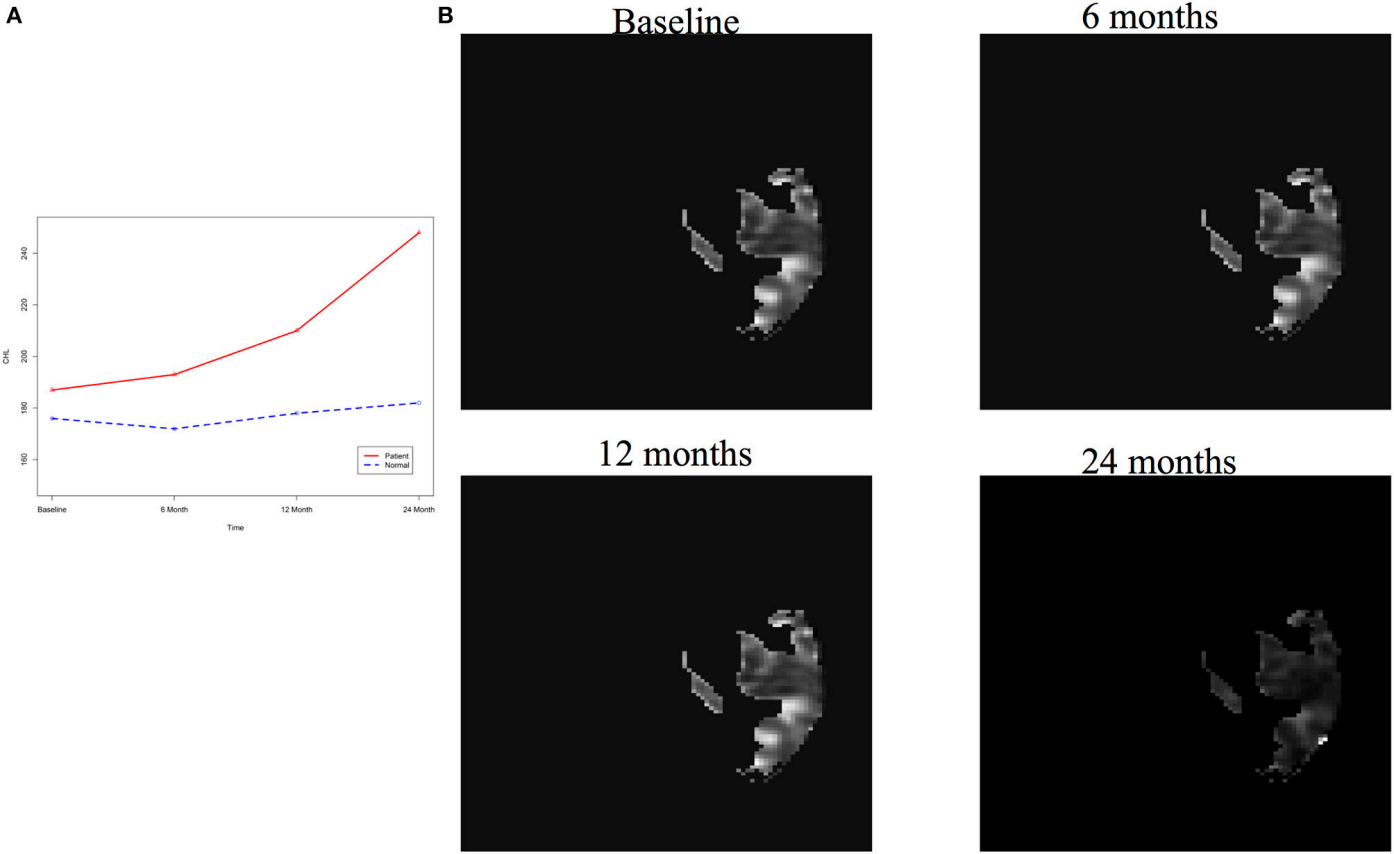
**(A)** AD and normal individuals' CHL curves. **(B)** Images of temporal L hippocampus region.

**Figure 14:**
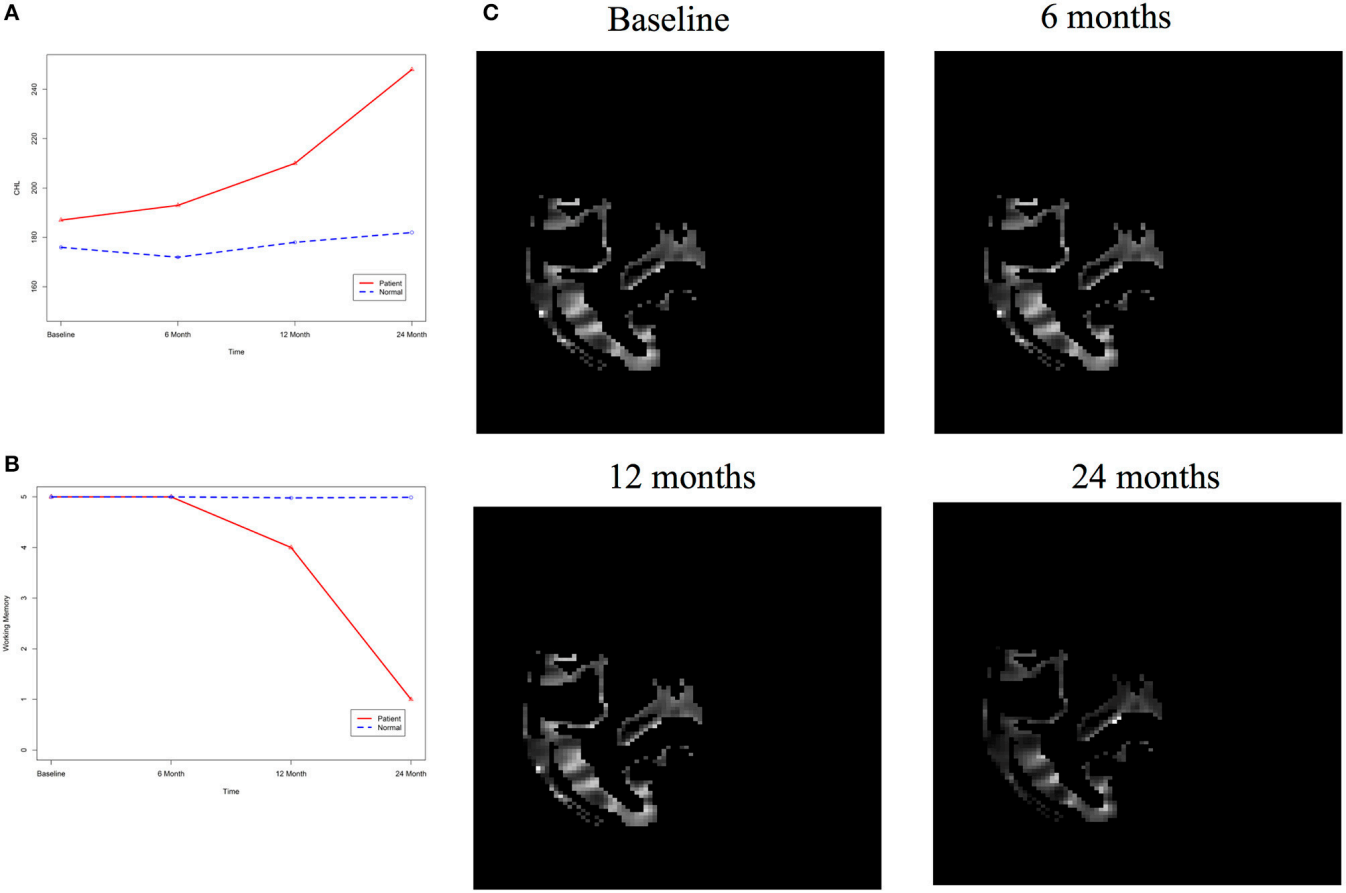
**(A)** AD and normal individuals' CHL curves. **(B)** AD and normal individuals' working memory. **(C)** Images of temporal R hippocampus region.

**Figure 15:**
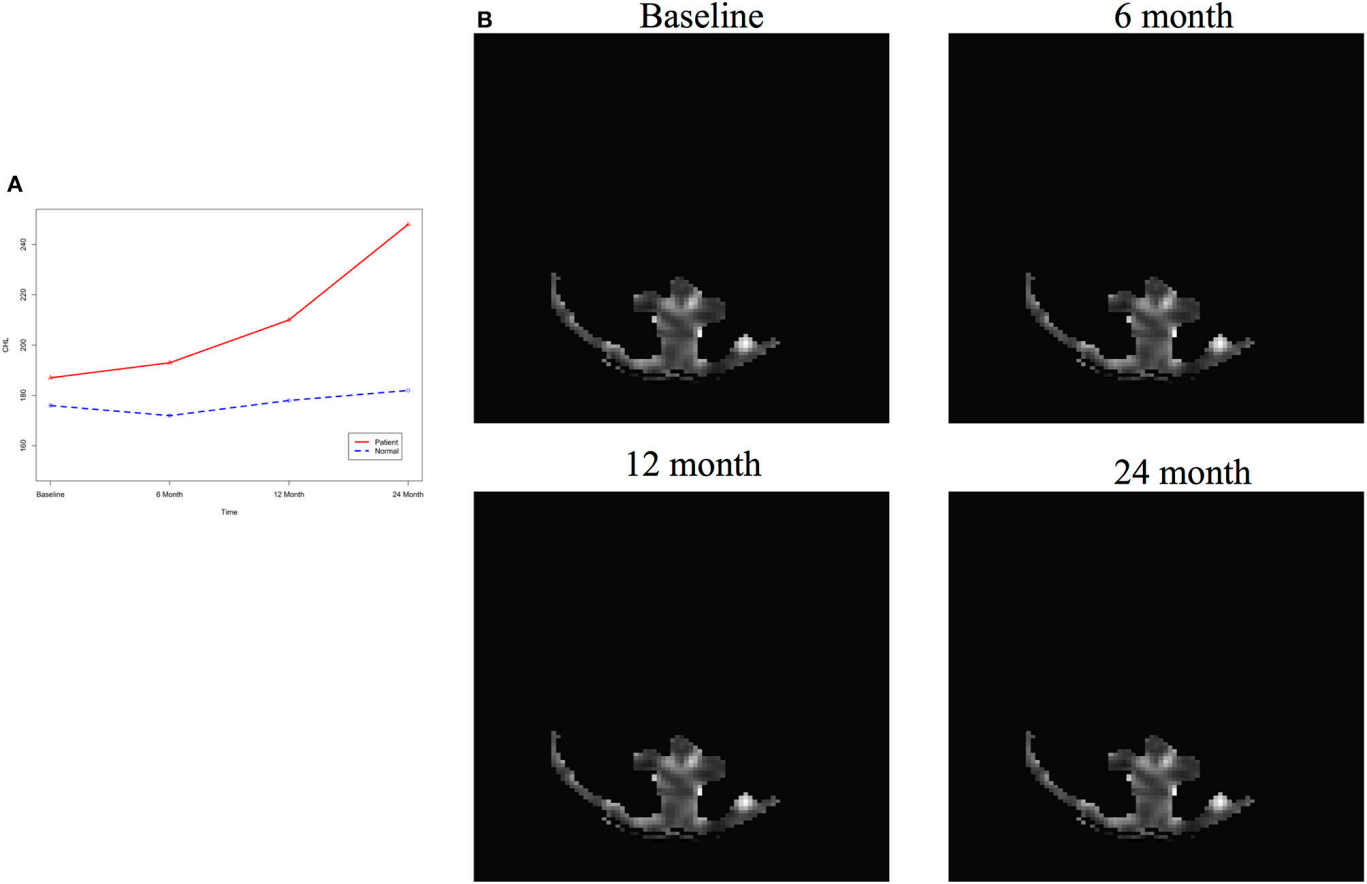
**(A)** AD and normal individuals' CHL curves. **(B)** Images of occipital lobe region.

After image imputation, DTI images at all four points and cholesterol and working memory of 91 individuals were available. The DTI images were segmented into 19 brain regions using the Super-voxel method (Achanta et al., 2012). Three-dimensional functional principal component analysis was used to summarize imaging signals in the brain region (Lin et al., 2015). The ANMs were used to infer causal relationships between cholesterol, or working memory and image where only first FPC score (accounting for more than 95% of the imaging signal variation in the segmented region) was used to present the imaging signals in the segmented region. Table 2 presented *P*-values for testing causation (cholesterol → image variation) and association of cholesterol with images of 19 brain regions where the canonical correlation method was used to test association (Lin et al., 2017). Two remarkable features emerged. First, we observed both causation and association of cholesterol with imaging signal variation at 24 months in the temporal L hippocampus (*P*-value for causation < 0.00013, *P*-value for association < 0.00007) and temporal R hippocampus regions (*P*-value for causation < 0.0165, *P*-value for association < 0.0044), and only association of cholesterol with imaging signal variation at 12 months in the temporal L region (*P*-value for causation < 0.5262, *P*-value for association < 0.0038). Figures 13A,B presented the curves of cholesterol level of an AD patient and average cholesterol level of normal individuals, and images at baseline, 6, 12, and 24 months of the temporal L hippocampus of an individual with AD diagnosed at 24 months time point, respectively. Figures 14A,C presented the curves of cholesterol level of an individual with AD diagnosed at 24 months' time point and average cholesterol levels of normal individuals, and images at baseline, 6, 12, and 24 months of the Temporal R regions of an individual with AD diagnosed at 24 months' time point, respectively. Figures 13, 14 showed that images of the temporal L hippocampus and Temporal R regions at 24 months became black, which indicated that temporal L hippocampus and temporal R regions were damaged by the high cholesterol. Second, we observed only association of cholesterol with imaging signal variation at 12 and 24 months in the Occipital_Mid brain region (*P*-value < 0.0003 at 12 months, *P*-value < 0.00004 at 24 months), but no causation (*P*-value < 0.6794 at 12 months, *P*-value < 0.1922 at 24 months). Figure 15 showed images of the occipital lobe region. We observed that there was no significant imaging signal variation in the occipital lobe region. This strongly demonstrates that association may not provide information on unraveling mechanism of complex phenotypes.

**Table 2:** *P-*values for assessing association and causal relationships between the cholesterol and brain region.

	**Baseline**		**6 Months**		**12 Months**		**24 Months**	
	**Causal**	**Association**	**Causal**	**Association**	**Causal**	**Association**	**Causal**	**Association**
Frontal_Inf_R	0.5699	0.4318	0.2927	0.9390	0.2169	0.7145	0.6624	0.1580
Frontal_Sup_Mid_L	0.4061	0.5539	0.0203	0.0301	0.6905	0.8670	0.3316	0.9664
Insula_L	0.9274	0.4602	0.2766	0.3102	0.5396	0.2724	0.7734	0.6819
Fusiform_L	0.3253	0.6601	0.8358	0.1778	0.5720	0.6238	0.8411	0.4510
Insula_R	0.3853	0.2367	0.6093	0.8874	0.0109	0.1218	0.2575	0.1832
Temporal_R	0.3740	0.7487	0.2997	0.3214	0.2813	0.8856	0.0165	0.0044
Occipital_Mid	0.7275	0.3344	0.8082	0.4159	0.6794	0.0003	0.1922	0.00004
Temporal_L	0.1455	0.4873	0.5384	0.9752	0.5262	0.0038	0.0001	0.0001
Frontal_L_R	0.1673	0.9822	0.8928	0.9269	0.3784	0.4762	0.5832	0.8093
Frontal & Temp_L	0.6067	0.4698	0.9643	0.3847	0.2945	0.9249	0.5057	0.1937
Lingual	0.2625	0.5307	0.8354	0.0834	0.7238	0.8036	0.2230	0.5510
Cingulum	0.6232	0.6483	0.3061	0.1381	0.0587	0.7611	0.3581	0.6024
Precentral_R	0.7113	0.4946	0.7263	0.0948	0.1565	0.6969	0.5169	0.6388
Frontal_Inf_L	0.9167	0.9260	0.5886	0.0138	0.3091	0.0929	0.3568	0.7203
Occipital	0.2444	0.3753	0.0782	0.9927	0.8490	0.2909	0.7388	0.4617
Precuneus	0.8480	0.2492	0.4183	0.9418	0.7208	0.5096	0.9071	0.8899
SMP	0.9866	0.1630	0.4416	0.6642	0.1175	0.3797	0.9788	0.3388
Precentral_L	0.6825	0.7937	0.4142	0.0759	0.9402	0.5150	0.5254	0.9770
Precentral_R	0.0488	0.4103	0.9759	0.9831	0.7251	0.9000	0.5008	0.0105

In our phenotype-image studies, we also identified causal relationships between working memory and activities of the temporal R (hippocampus) at 24 months with *P*-value < 0.00014) (image → working memory), but identified no association of working memory with imaging signal variation in the temporal R (hippocampus) region (*P*-value < 0.5904) (Table 3). Figure 14C showed the weak imaging signal or decreased neural activities in the temporal R (hippocampus) region at 24 months and Figure 14B showed lower working memory measure of an AD patient than the average working memory measurements of normal individuals at 24 months. This demonstrated that the decreased neural activities in the temporal R (hippocampus) region deteriorated working memory of the AD patient. This result provided evidence that causation may be identified in the absence of association signals. These observations can be confirmed from the literature. It was reported that cholesterol level impacted the brain white matter connectivity in the temporal gyrus (Haltia et al., 2007) and was related to AD (Sjögren and Blennow, 2005; Teipel et al., 2006). Abnormality in working memory was observed in patients with temporal lobe epilepsy (Stretton et al., 2013).

**Table 3:** *P*-values for assessing association and causal relationships between the working memory and brain region.

	**Baseline**		**6 Months**		**12 Months**		**24 Months**	
	**Causal**	**Association**	**Causal**	**Association**	**Causal**	**Association**	**Causal**	**Association**
Frontal_Inf_R	0.7515	0.6348	0.4857	0.5088	0.3709	0.5807	0.5028	0.0572
Frontal_Sup_Mid_L	0.2022	0.2877	0.0187	0.8929	0.2355	0.8327	0.4114	0.7976
Insula_L	0.0300	0.5539	0.4928	0.1057	0.8959	0.5846	0.6212	0.0332
Fusiform_L	0.3244	0.5135	0.0931	0.0503	0.0617	0.9162	0.6927	0.0741
Insula_R	0.2212	0.9885	0.7729	0.6777	0.5171	0.1434	0.7416	0.4923
Temporal_R	0.9042	0.5224	0.9641	0.6987	0.2813	0.0939	0.0001	0.5904
Occipital_Mid	0.8350	0.4884	0.0309	0.7277	0.6280	0.9993	0.2067	0.4716
Temporal_L	0.9491	0.8716	0.1052	0.4597	0.0001	0.0006	0.0001	0.5836
Frontal_L_R	0.8957	0.0212	0.2522	0.5165	0.2658	0.7134	0.1474	0.1720
Frontal & Temp_L	0.9189	0.3919	0.7792	0.1148	0.3951	0.3585	0.7691	0.7355
Lingual	0.4241	0.3219	0.4952	0.5941	0.1707	0.8981	0.8382	0.6736
Cingulum	0.5063	0.5778	0.0383	0.9534	0.5947	0.3123	0.1482	0.6307
Precentral_R	0.1398	0.2945	0.9875	0.5693	0.3247	0.7966	0.7323	0.7358
Frontal_Inf_L	0.8985	0.0989	0.2982	0.3727	0.8644	0.0363	0.9291	0.9581
Occipital	0.3828	0.8736	0.5267	0.8378	0.4624	0.1352	0.6937	0.1991
Precuneus	0.7215	0.8909	0.1169	0.5417	0.0406	0.6599	0.0429	0.9704
SMP	0.0900	0.7818	0.9407	0.6380	0.4428	0.3417	0.3151	0.8178
Precentral_L	0.9660	0.7217	0.6289	0.6630	0.8759	0.5526	0.8848	0.1713
Precentral_R	0.4051	0.3829	0.4783	0.5286	0.6365	0.0569	0.9260	0.5996

Next we investigate two examples from the gold-standard data set in (Mooij et al., 2016) to evaluate performance. The first dataset was collected at 349 weather stations in Germany from 1961 to 1990. Let *X* be altitude and *Y* be temperature. Meteorology assumes that places with higher altitude tend to be colder than those with lower altitude (roughly 1 centigrade per 100 meter). There is no doubt that altitude is the cause and temperature the effect, so ground truth is *X* → *Y*. *P*-value of using the ANMs and permutation test for detecting the causation was 0.001.

The second dataset was Old Faithful geyser data. Old Faithful is a hydrothermal geyser in Yellowstone National Park in the state of Wyoming, USA. Each observation corresponds to a single eruption. The data consists of 194 samples, and was collected in a single continuous measurement from August 1 to August 15, 1985. Let *X* be duration of eruption in minutes and *Y* be time to the next eruption in minutes. It is commonly accepted that the time interval between the current and the next eruption is an effect of the duration of the current eruption, so ground truth is *X* → *Y*. *P*-value of using the ANMs and permutation test for detecting the causation was 0.003. Both examples demonstrated that the ANMs and permutation test were able to detect causation between two variables.

## Discussion

The major purpose of this paper is to address several issues for shifting the paradigm of genetic analysis from association analysis to causal inference and to focus on causal discovery between two variables. The first issue is the basic principles for causal inference from observational data only. Typical methods for unraveling cause and effect relationships are interventions and controlled experiments. Unfortunately, the experiments in human genetics are unethical and technically impossible. In the past decade, the new principles for causal inference from pure observational data have been developed. The philosophical causal principle assumes that nature consists of autonomous and independent causal generating process modules and attempts to replace causal faithfulness by the assumption of Independence of Cause and Mechanism (ICM). In other words, causal generating processes of a system's variables are independent. If we consider two variables, the ICM states that distribution of cause and conditional distribution of effect given the cause are independent.

The second issue is how to measure independence (or dependence) between two distributions. Statistics only provides tools for measuring independence between two random variables. There are no measures or statistics to test independence between two distributions. Therefore, we introduce algorithmic information theory that can offer notion and mathematical formulation of independence between two distributions or independence of mechanisms. We use algorithmic mutual information to measure independence between two distributions which can be used to assess causal relationships between two variables. Algorithmically independent conditional implies that the joint distribution has a shorter description in causal direction than in non-causal direction.

The third issue is to develop causal models that can easily assess algorithmic independent conditions. The algorithmic independent condition states that the direction with the lowest Kolmogorov complexity can be identified to be the most likely causal direction between two random variables. However, it is well-known that the Kolmogorov complexity is not computable (Budhathoki and Vreeken, 2017). Although stochastic complexity was proposed to approximate Kolmogorov complexity via the Minimum Description Length (MDL) principle, it still needs heavy computations. The ANM was developed as practical causal inference methods to implement algorithmically independent conditions. We showed that algorithmic independence between the distribution of cause *X* and conditional distribution *P*_*Y*|*X*_ of effect given the cause is equivalent to the independence of two random variables *X* and *E*_*Y*_ in the ANM.

The fourth issue is the development of test statistics for bivariate causal discovery. The current ANM helps to break the symmetry between two variables *X* and *Y*. Its test statistics are designed to identify causal directions: *X* → *Y* or *Y* → *X*. Statistics and methods for calculation of *P*-values for testing the causation between two variables have not been developed. To address this issue, we have developed a new statistic to directly test for causation between two variables and a permutation method for the calculation of *P*-value of the test.

The fifth issue is the power of the ANM. The challenge arising from bivariate causal discovery is whether the ANM has enough power to detect causation between two variables. To investigate their feasibility for causal inference, the ANMs were applied to simulation data. We considered three non-linear functions: quadratic, exponential, and logarithm functions and two random noise variables: normal and t distribution. We showed that the ANM had reasonable power to detect existence of causation between two variables. To further evaluate its performance, the ANM was also applied to reconstruction of the Wnt pathway using gene expression data. The results demonstrated that the ANM had higher power to infer gene regulatory networks than six other statistical methods using KEGG pathway database as gold standard.

The sixth issue is how to distinguish association from causation. In everyday language, correlation and association are used interchangeably. However, correlation and association are different terminologies. Correlation is to characterize the trend pattern between two variables, particularly; the Pearson correlation coefficient measures linear trends, while association characterizes the simultaneous occurrence of two variables. The widely used notion of association often indicates the linear correlation. When two variables are linearly correlated we say that there is association between them. Pearson correlation or its equivalent, linear regression is often used to assess association. Causation between two variables is defined as independence between the distribution of cause and conditional distribution of the effect, given cause. In the non-linear ANM, the causal relation is assessed by testing independence between the cause variable and residual variable. We investigated the relationships between causation and association (linear correlation). Some theoretical analysis and real trait-imaging data analysis showed that there were three scenarios: (1) presence of both association and causation between two variables, (2) presence of association, while absence of causation, and (3) presence of causation, while lack of association in causal analysis.

Finally, in real imaging data analysis, we showed that causal traits change the imaging signal variation in the brain regions. However, the traits that were associated with the imaging signal in the brain regions did not change imaging signals in the region at all.

The experiences in association analysis in the past several decades strongly demonstrate that association analysis is lack of power to discover the mechanisms of the diseases and provide powerful tools for medicine. It is time to shift the current paradigm of genetic analysis from shallow association analysis to more profound causal inference. Transition of analysis from association to causation raises great challenges. The results in this paper are considered preliminary. A large proportion of geneticists and epidemiologists have serious doubt about the feasibility of causal inference in genomic and epigenomic research. Causal genetic analysis is in its infantry. The novel concepts and methods for causal analysis in genomics, epigenomics, and imaging data analysis should be developed in the genetic community. Large-scale simulations and real data analysis for causal inference should be performed. We hope that our results will greatly increase the confidence in genetic causal analysis and stimulate discussion about whether the paradigm of genetic analysis should be changed from association to causation or not.

## Author contributions

RJ: Perform data analysis and write paper; NL and ZH: Perform data analysis; DB: Provide data and result interpretation; LJ: Design project; MX: Design project and write paper.

### Conflict of interest statement

The authors declare that the research was conducted in the absence of any commercial or financial relationships that could be construed as a potential conflict of interest.
