# Supplementary material for: Bivariate Causal Discovery and Its Applications to Gene Expression and Imaging Data Analysis
Source: Front Genet. 2018 Aug 31;9:347. doi: 10.3389/fgene.2018.00347 (PMC6127271; doi:10.3389/fgene.2018.00347)
Supplement: Supplementary file 1 [file Table_1.DOCX]

| Table S1. Type 1 error rates of the ANMs for testing causation, assuming no association. | | | |
| --- | --- | --- | --- |
|  | Number of Samples | | |
| Nominal Levels | 500 | 1000 | 2000 |
| 0.05 | 0.033 | 0.051 | 0.043 |
| 0.01 | 0.006 | 0.005 | 0.01 |
