# Supplementary material for: Bivariate Causal Discovery and Its Applications to Gene Expression and Imaging Data Analysis
Source: Front Genet. 2018 Aug 31;9:347. doi: 10.3389/fgene.2018.00347 (PMC6127271; doi:10.3389/fgene.2018.00347)
Supplement: Supplementary file 2 [file Table_2.DOCX]

| Table S2. Type 1 error rates of the ANMs for testing causation in the presence of association. | | | |
| --- | --- | --- | --- |
|  | Number of Samples | | |
| Nominal Levels | 500 | 1000 | 2000 |
| 0.05 | 0.044 | 0.048 | 0.050 |
| 0.01 | 0.011 | 0.011 | 0.011 |
