# Supplementary material for: Bivariate Causal Discovery and Its Applications to Gene Expression and Imaging Data Analysis
Source: Front Genet. 2018 Aug 31;9:347. doi: 10.3389/fgene.2018.00347 (PMC6127271; doi:10.3389/fgene.2018.00347)
Supplement: Supplementary file 3 [file Table_3.DOCX]

| Table S3. Power of the ANMs for detecting causation between two variables. | | | | |  |
| --- | --- | --- | --- | --- | --- |
| Significance Level | Number of Samples | | | | |
|  | 200 | 500 | 1000 | 2000 | 5000 |
| 0.05 | 0.3616 | 0.4833 | 0.5629 | 0.5997 | 0.6412 |
| 0.01 | 0.2066 | 0.3556 | 0.4382 | 0.4762 | 0.5241 |
