# Supplementary material for: Bivariate Causal Discovery and Its Applications to Gene Expression and Imaging Data Analysis
Source: Front Genet. 2018 Aug 31;9:347. doi: 10.3389/fgene.2018.00347 (PMC6127271; doi:10.3389/fgene.2018.00347)
Supplement: Supplementary file 4 [file Data_Sheet_1.docx]

**Supplementary Note A**

A Turing machine is a hypothetical machine developed by Alan Turing in 1936. Turing machine is designed to simulate any computer algorithm, no matter how complicated it is (Ashrafian et al. 2015). Consider a universal Turing Machine $T$. For any binary string $s$, we define Kolmogorov complexity $K_{T}(s)$ as the length of the shortest program that generates $s$, denoted as $s^{*}$, using universal prefix Turing machine $T$ that outputs $s$ and then stops (Peters et al. 2017; Kolmogorov 1965). Therefore, we have $K_{T}\left( s \right)=|s^{*}|$, where $|.|$ denotes the number of bits of a binary string. Intuitively, the Kolmogorov complexity measures the minimal amount of information required to generate $s$ by any effective process. Similar to conditional probability, we can also define conditional Kolmogorov complexity. The conditional Kolmogorov complexity $K(t|s)$ of string $t$ given $s$, is defined as the length of the shortest program that can generate $t$ from $s$ and then stops. The Kolmogorov complexity $K(t,s)$ of the concatenation of two strings $t$ and $s$ is defined as the length of the shortest program that generate string $t's$ , where $t^{'}$is the prefix code of $t$.

Now we introduce “additivity of complexity” property. It can be shown that (Grunwald and Vitanyi 2004):

$K\left( t,s \right)=K\left( t \right)+K(s|t^{*})$, (A1)

where $t^{*}$ denotes the first shortest prefix program that generates $t$ and then stops and is in general uncomputable.

Algorithmic mutual information is defined as

$I\left( s:t \right)=K\left( s \right)-K(s|t^{*})$. (A2)

Substituting $K(s|t^{*})$ in equation (A1) into equation (A2), we obtain

$I\left( s:t \right)⩲K\left( s \right)+K\left( t \right)-K(s,t)$, (A3)

where the symbol ⩲ implies that the equation can hold for up to constants. Equation (A3) states that this information is symmetrical: $I\left( s:t \right)=I(t:s)$. Therefore, $I(s:t)$ is called algorithmic mutual information between $s$ and $t$. The algorithmic mutual information quantifies the amount of information two strings or objects have in common, or the amount of bits saved when compressing $s, t$ jointly rather than compressing $s, t$ independently.

Similar to mutual information $I\left( s;t \right)$ between two random variables where mutual information of zero implies independence of two variables, the algorithmic mutual information of zero $I(s:t)$ indicates algorithmically independence of two distributions of random variables. We also can define algorithmic conditional mutual information as

$I\left( s:t|z \right)⩲K\left( s|z \right)+K\left( t|z \right)-K(s,t|z)$. (A4)

In statistics, although dependence between two random variables can be measured, there are no measures to quantify dependence between two distributions. We use algorithmic mutual information to measure independence between two distributions which can be used to assess causal relationships between two variables. Consider two variables $X$ and $Y$ and assume $X$ causes $Y$ ($X\to Y$). Let the marginal distribution of cause $X$ and conditional distribution of effect $Y$given $X$ be $P_{X}$and $P_{Y|X}$, respectively. The independence of cause and mechanism (ICM) states that the distributions $P_{X}$and $P_{Y|X}$ are independent and hence $P_{X}$and $P_{Y|X}$ are algorithmically independent, which implies that their algorithmic mutual information should be equal to zero (Peters et al. 2017):

$I\left( P_{X}:P_{Y|X} \right)⩲0$, (A5)

or , equivalently,

$K(P_{X,Y})⩲K(P_{X}$)$+K(P_{Y|X})$. (A6)

In other words, distributions $P_{X}$and $P_{Y|X}$ have no common information. If $X$ causes $Y$, then the conditional distribution $P_{Y|X}$ of the effect $Y$ given cause $X$ contains no information about cause $X$. Thus, the algorithmic mutual information can be used to infer whether $X\to Y$ or $Y\to X$. If $I\left( P_{X}:P_{Y|X} \right)<I(P_{Y}:P_{X|Y})$ then $X\to Y$. Similarly, if $I\left( P_{X}:P_{Y|X} \right)>I(P_{Y}:P_{X|Y})$ then $Y\to X$. Cause and effect cannot be identified from their joint distribution. Cause and effect are asymmetric. The joint distribution is symmetric. It can be factorized to $P_{X,Y}=P_{X}P_{Y|X}=P_{Y}P_{X|Y}$.

**Supplementary Note B**

We show that for every joint distribution $P_{X,Y}$ of real-valued variables $X$ and $Y$, there is a nonlinear model:

$Y=f_{Y}\left( X,N_{Y} \right), X⫫N_{Y}$,

where $f_{Y}$ are functions and $N_{Y}$ is a real-valued noise variables.

Proof.

Define the conditional cumulative distribution function:

$F_{Y|X}\left( y \right)=P(Y\leq y|X=x)$ (B1)

and let

$N_{Y}=F_{Y|X}\left( y \right)$. (B2)

Define its inverse function

$F_{Y|x}^{-1}\left( n_{Y} \right):=$inf $\{z\in R: F_{Y|x}(z)\geq n_{Y}\}$. (B3)

Define function

$f_{Y}\left( x,n_{Y} \right):=F_{Y|x}^{-1}\left( n_{Y} \right)$. (B4)

Now we make changes of variables:

$x=x$ (B5)

and

$y=f_{Y}\left( x,n_{Y} \right)$. (B6)

The Jacobian matrix of the transformation is given by

$J=\left| \begin{matrix} 1 & 0 \\ 0 & \frac{1}{{F^{'}}_{Y|x}(y)} \end{matrix} \right|=\frac{1}{{F^{'}}_{Y|x}(y)}$, (B7)

where $P_{Y|x}\left( y \right)={F^{'}}_{Y|x}(y$).

Using equation (B2), we obtain that $N_{Y}$ is uniformly distributed on [0, 1]. If we assume that $N_{Y}$ is independent of $X$, then using the distribution transform theorem, we obtain

$P_{Y,X}=\frac{P_{X}}{J}=P_{X}P_{Y|X}$. (B8)

**Supplementary Note C**

To illustrate application of the algorithmic mutual information, we show that independence of cause and mechanism will imply that the cause $X$ and error $E_{Y}$ in the nonlinear function model (1) are independent.

Independence of cause and mechanism states that the conditional distribution $P_{Y|X}$ contains no information about the distribution of causal $P_{X}$. In other words, $P_{X}$ and $P_{Y|X}$ are algorithmically independent:

$I(P_{X}:P_{Y|X})⩲0$. (C1)

Assume a nonlinear function model:$Y=f\left( X \right)+E_{Y}$, but we do not assume that $X$ and $E_{Y}$ are independent. We now show that independence of cause and mechanism implies that $X$ and $E_{Y}$ are independent in the ANM (1).

The principle independence of cause and mechanism implies that $P_{Y|X} ⩲ P_{E_{Y}}$. Therefore, from equation (C1) we obtain

$I(P_{X}:P_{E_{Y}})⩲0$, (C2)

which implies

$I\left( X;E_{Y} \right)=0$. (C3)

Mutual information of zero between the cause $X$and residual variable $E_{Y}$ shows that $X$ and $E_{Y}$ are independent. It is also well known that if $X$ and $E_{Y}$ are independent, then $I(P_{X}:P_{E_{Y}})⩲0$ or $I(P_{X}:P_{Y|X})⩲0$ (Janzing and Schölkopf 2010). Therefore, algorithmic independence between the distribution of cause $X$ and conditional distribution $P_{Y|X}$ of effect given the cause is equivalent to the independence of two random variables $X$ and $E_{Y}$ in the ANM.
