# Supplementary figures and images for: Bivariate Causal Discovery and Its Applications to Gene Expression and Imaging Data Analysis
Source: Front Genet. 2018 Aug 31;9:347. doi: 10.3389/fgene.2018.00347 (PMC6127271; doi:10.3389/fgene.2018.00347)

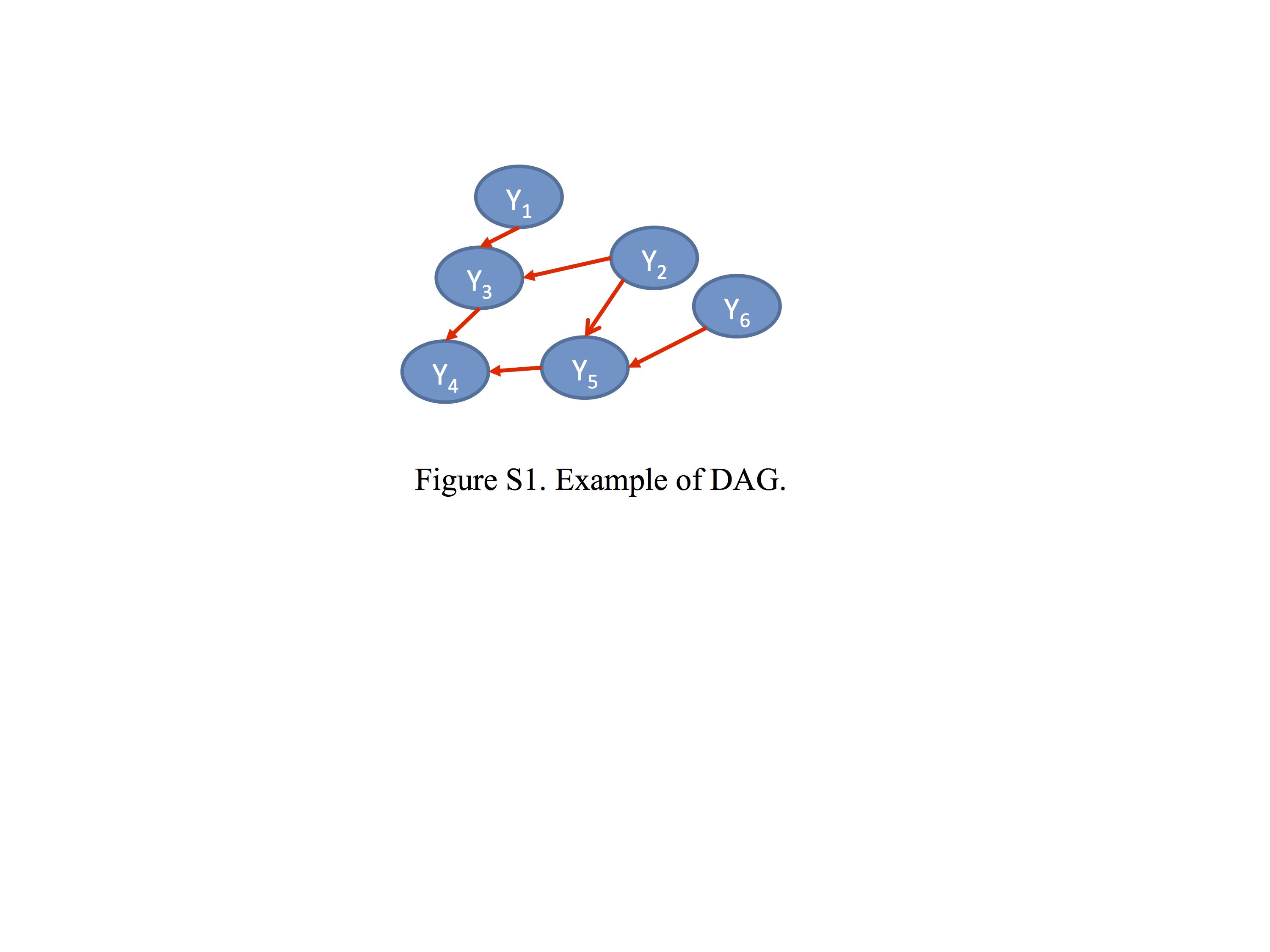

Supplement: Supplementary file 5 [file Image_1.TIFF]

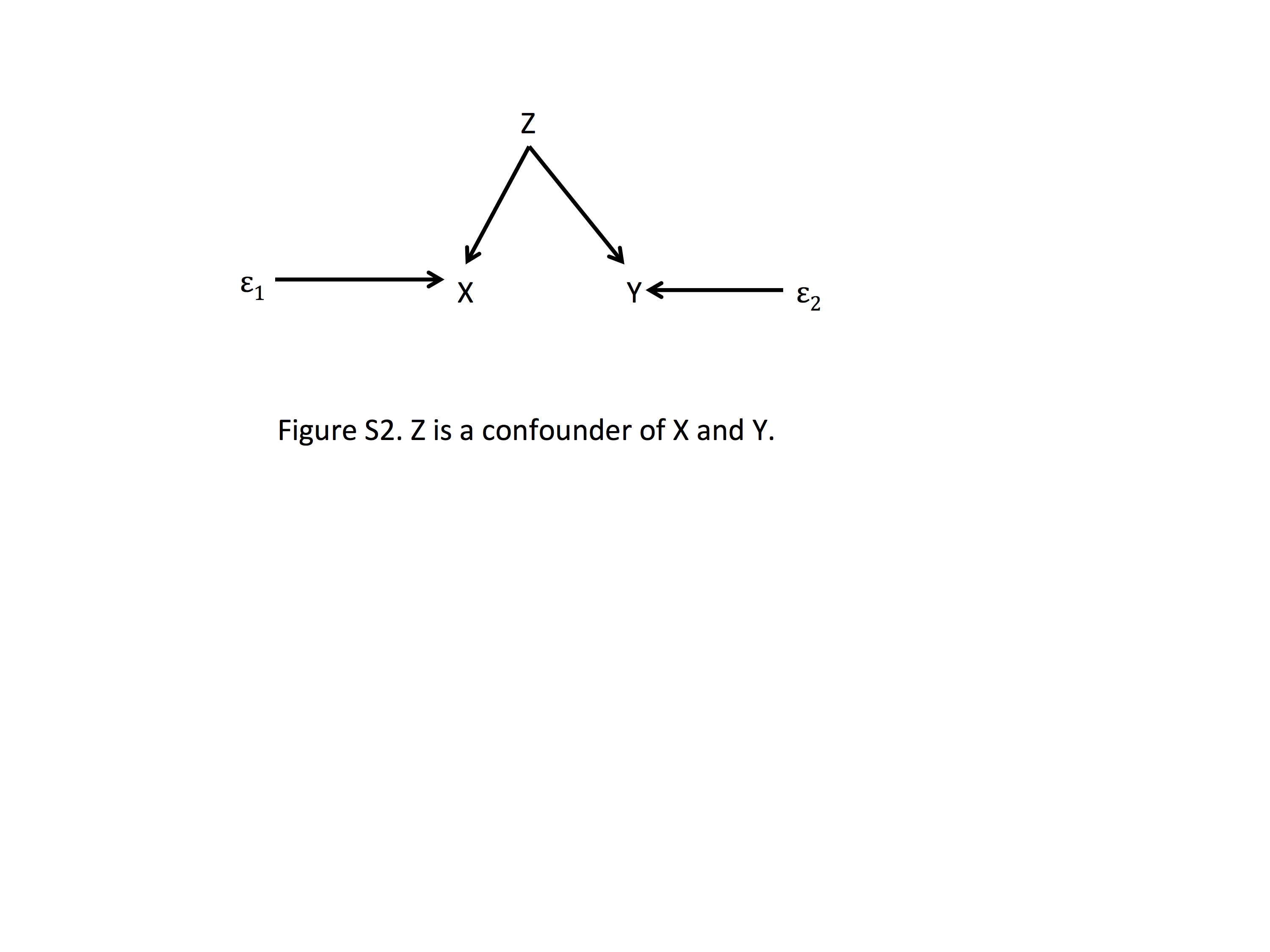

Supplement: Supplementary file 6 [file Image_2.TIFF]
